# Pharmacokinetics and Pharmacodynamics with Extended Dosing of CC-486 in Patients with Hematologic Malignancies

**DOI:** 10.1371/journal.pone.0135520

**Published:** 2015-08-21

**Authors:** Eric Laille, Tao Shi, Guillermo Garcia-Manero, Christopher R. Cogle, Steven D. Gore, Joel Hetzer, Keshava Kumar, Barry Skikne, Kyle J. MacBeth

**Affiliations:** 1 Celgene Corporation, Summit, New Jersey, United States of America; 2 Department of Leukemia, University of Texas, MD Anderson Cancer Center, Houston, Texas, United States of America; 3 Division of Hematology/Oncology, University of Florida, Gainesville, Florida, United States of America; 4 Yale Cancer Center, New Haven, Connecticut, United States of America; University Hospital of Salamanca, SPAIN

## Abstract

CC-486 (oral azacitidine) is an epigenetic modifier in development for patients with myelodysplastic syndromes and acute myeloid leukemia. In part 1 of this two-part study, a 7-day CC-486 dosing schedule showed clinical activity, was generally well tolerated, and reduced DNA methylation. Extending dosing of CC-486 beyond 7 days would increase duration of azacitidine exposure. We hypothesized that extended dosing would therefore provide more sustained epigenetic activity. Reported here are the pharmacokinetic (PK) and pharmacodynamic (PD) profiles of CC-486 extended dosing schedules in patients with myelodysplastic syndromes (MDS), chronic myelomonocytic leukemia (CMML) or acute myeloid leukemia (AML) from part 2 of this study. PK and/or PD data were available for 59 patients who were sequentially assigned to 1 of 4 extended CC-486 dosing schedules: 300mg once-daily or 200mg twice-daily for 14 or 21 days per 28-day cycle. Both 300mg once-daily schedules and the 200mg twice-daily 21-day schedule significantly (all *P* < .05) reduced global DNA methylation in whole blood at all measured time points (days 15, 22, and 28 of the treatment cycle), with sustained hypomethylation at cycle end compared with baseline. CC-486 exposures and reduced DNA methylation were significantly correlated. Patients who had a hematologic response had significantly greater methylation reductions than non-responding patients. These data demonstrate that extended dosing of CC-486 sustains epigenetic effects through the treatment cycle.

**Trial Registration:**

ClinicalTrials.gov *NCT00528983*

## Introduction

Altered methylation of DNA is common in cancers, including myelodysplastic syndromes (MDS), with widespread hypermethylation of CpG islands in promoter regions of genes involved in normal cell cycle regulation, differentiation, and apoptosis [1–4]. Therapeutic targeting of aberrantly hypermethylated genes may restore cancer-suppressing functions to cells. Azacitidine is a cytidine analog epigenetic modifier that is incorporated into DNA and RNA [5–8]. Once incorporated into DNA, azacitidine inactivates DNA methyltransferases (DNMTs) [9, 10] causing DNA hypomethylation by passive methylation loss during cell division [11, 12]. Azacitidine may also have an effect on ribonucleotide reductase, reducing the conversion of the RNA pathway congener form, 5-azacitidine-diphosphate, to the DNA congener precursor form, 5-deoxy-azacitidine-diphosphate [13]. Additional mechanisms of azacitidine activity may be mediated via incorporation into newly synthesized RNA, which can account for 65% to 90% of the azacitidine incorporated into cellular nucleic acid [5, 14]. Azacitidine is believed to exert its antineoplastic effects by both reducing DNA methylation and inducing cytotoxicity in abnormal hematopoietic cells in the bone marrow [5, 15–19]; however, the mechanisms linked to azacitidine clinical efficacy have not been completely elucidated.

Given the short plasma half-life [20] and S-phase-restricted DNA incorporation of azacitidine, [5, 17, 21] drug exposure time is likely to influence the number of diseased target cells affected, especially in diseases with a low S-phase fraction, such as MDS [22]. Optimal drug activity may require an increased window of exposure to malignant cells to increase the opportunity for cycling cells to incorporate drug [7, 23]. The importance of chronic exposure is consistent with the clinical observation that optimal therapeutic effects of hypomethylating agents used for treatment of MDS require multiple treatment cycles to manifest [24–26]. The approved azacitidine dosing regimen is 75 mg/m^2^ administered subcutaneously (SC) or intravenously (IV) over 7 consecutive days of repeated 28-day cycles, leaving 21 drug-free days each cycle. Extending the duration of azacitidine exposure using CC-486, the oral formulation of azacitidine, may provide the ability to achieve and sustain the epigenetic activity of azacitidine beyond what is practically achievable with the parenteral formulation.

Part 1 of a two-part, multicenter, dose-finding study showed CC-486 to be bioavailable, safe, and clinically active, with a maximally tolerated dose of 480 mg/day, when administered once daily for 7 days of repeated 28-day cycles in patients with MDS, chronic myelomonocytic leukemia (CMML), and acute myeloid leukemia (AML) [20]. A lesser degree of hypomethylation was observed at mid-cycle with the 7-day CC-486 regimen compared with SC azacitidine, and methylation level returned to baseline at cycle end with both regimens. We hypothesized that extending the CC-486 treatment schedule to 14 or 21 days per 28-day treatment cycle would provide more sustained reduction of DNA methylation by increasing the likelihood that cycling diseased progenitor cells would be exposed to drug. Reported here are the pharmacokinetic (PK) and pharmacodynamic (PD) profiles of extended CC-486 dosing schedules from part 2 of this study.

## Methods

This open-label study is registered at ClinicalTrials.gov (*NCT00528983*). All procedures pertaining to the conduct, evaluation, and documentation of this study were in accordance with Good Clinical Practice (GCP), as described in the International Conference on Harmonization (ICH) Guideline E6, and complied with general ethical principles outlined in the Declaration of Helsinki. The institutional review boards (IRBs) that approved the study are listed in S1 Table. All patients provided written informed consent before participation. All authors had access to all study data. The first patient was screened on September 6, 2007, and the last patient completed the main study phase on July 31, 2013. Patients benefiting from treatment could enroll in an optional extension phase; at the time of manuscript submission, the extension phase is ongoing. Study data were analyzed by statisticians and scientists from Celgene Corporation (Summit, NJ). The authors confirm that all ongoing and related Celgene-sponsored trials for CC-486 are registered.

### Study design and patients

Eligible patients were aged 18 years or older, with Eastern Cooperative Oncology Group (ECOG) performance status score of 0 to 2, and a diagnosis of MDS, CMML, or AML according to the WHO classification system [27]. For patients with AML, eligibility was limited to those for whom curative measures were not available or no longer effective. Exclusion criteria included diagnosis of acute promyelocytic leukemia, any previous treatment with hypomethylating agents, anticancer treatments within 21 days before initiating study drug, and incomplete recovery from toxicity from previous treatments. All patients were referred to study by a participating investigator.

Patients were sequentially assigned (not randomized), irrespective of underlying diagnosis, to receive CC-486 in 1 of 4 extended dosing schedules for repeated 28-day cycles: 300 mg once-daily for 14 or 21 days, or 200 mg twice-daily for 14 or 21 days. Initially, a cohort was considered complete when it comprised 6 patients, but the 14- and 21-day QD dosing cohorts were later expanded to include additional patients per protocol amendment. In the twice-daily dosing schedules, CC-486 doses were ingested 12 hours (± 30 minutes) apart. CC-486 was administered at study sites on clinic visits and self-administered when home. The decision to collect PK and PD samples was made prospectively, before the patient entered the screening phase of the study. All evaluable PK and PD data are included in analyses.

### PK assessments

PK parameters were evaluated from plasma samples drawn on day 1 and the final dosing day of cycle 1 (day 14 or day 21 for the 14-day and 21-day dosing schedules, respectively). Plasma was collected before dosing and post-dose at 0.25, 0.5, 1, 1.5, 2, 2.5, 3, 3.5, 4, 6, and 8 hours.

Plasma samples were analyzed using a validated proprietary high-performance liquid chromatography/tandem mass spectrometric method. PK parameters included maximum observed plasma concentration (C_max_), time of maximum observed plasma concentration (T_max_), area under the plasma concentration-time curve from zero extrapolated to infinity (AUC_∞_), terminal elimination half-life (t_1/2_), apparent total clearance (CL/F), and apparent volume of distribution (Vz/F). PK parameters were calculated using non-compartmental methods with Phoenix WinNonlin software (Pharsight Corp, Mountain View, CA).

### PD assessments

DNA methylation levels were measured to determine the DNA hypomethylating activity of CC-486 in a subgroup of patients (based on sample availability and DNA yield). In cycle 1, whole blood was collected during screening (baseline) and before drug administration on days (±1 day) 1, 15, 22, and 28 (cycle end). Whole blood samples for PD analyses were also collected on day 1 of subsequent cycles for assessment of correlations between PD measures and clinical responses (described below).

Genomic DNA was purified from each whole blood sample using the PAXgene Blood DNA System (Qiagen; Valencia, CA). Methylation profiling was performed using the Infinium HumanMethylation27 BeadArray (Illumina; San Diego, CA). DNA methylation level at each genomic locus is described as beta value and calculated as the ratio of the methylated signal to the combined locus signal by BeadStudio. Global DNA methylation scores (GDMS) were assigned to each sample by calculating the percentage of highly methylated (beta ≥ 0.7) loci. The 0.7 cut-off was chosen because the overall distribution of DNA methylation levels for loci on the array was clearly bimodal, with two peaks centered at approximately beta = 0.1 and 0.85. This cut-off value has also been used in other studies [28]. Wilcoxon signed-rank tests were used to determine the significance of GDMS changes throughout the 28-day dosing cycle. A Wilcoxon rank- sum test was used to determine the difference in changes in GDMS between cohorts for a given day of the dosing cycle. The limma package (version 3.12.3 [29]) from Bioconductor (version 2.11) was used to identify specific loci with significant methylation changes at each post-treatment time point versus baseline. Loci with false discovery rate (FDR)-adjusted *P* values ≤ .05 and an absolute change in beta value of > 0.1 were considered statistically significant. All statistical analyses were performed using R statistical software (R Foundation for Statistical Computing; Vienna, Austria; http://www.R-project.org) and were restricted to 26,486 autosomal loci. NextBio [30] was used to assess pathway enrichment in the gene lists with methylation levels affected by CC-486 treatment.

### PK/PD correlation

The correlation between azacitidine exposure (AUC) and methylation change was assessed by linear regression in patients for whom both PK data (on cycle 1 day 1) and PD data (on cycle 1 day 1 and days 15, 22, or cycle end) were available. For each patient, AUC_total_ (extrapolated cumulative azacitidine exposure for the entire cycle) was calculated by multiplying the AUC at cycle 1 day 1 by the total number of doses administered during the cycle.

### PD/efficacy correlation

To determine if changes in methylation were associated with clinical response, box-and-whisker plots and Wilcoxon rank sum test were used to compare the changes in GDMS at time points throughout cycle 1 and on day 1 of subsequent cycles between patients with a clinical response, per modified International Working Group (IWG) 2003 criteria (with modifications) for patients with AML, [31] or IWG 2006 criteria for patients with MDS or CMML, [32] and patients with no response. Responses included complete remission (CR), marrow CR, attainment of red blood cell (RBC) or platelet transfusion independence in patients who were transfusion dependent at baseline; or hematologic improvement (any cell lineage). RBC transfusion dependence was defined as ≥ 4 RBC units in the 56 days before cycle 1, and platelet transfusion dependence was defined as ≥ 2 platelet transfusions in the 56 days before cycle 1. RBC or platelet transfusion independence was defined as no transfusions in any 56 consecutive-day period on-treatment.

## Results

### Patient disposition

**Fig 1:**
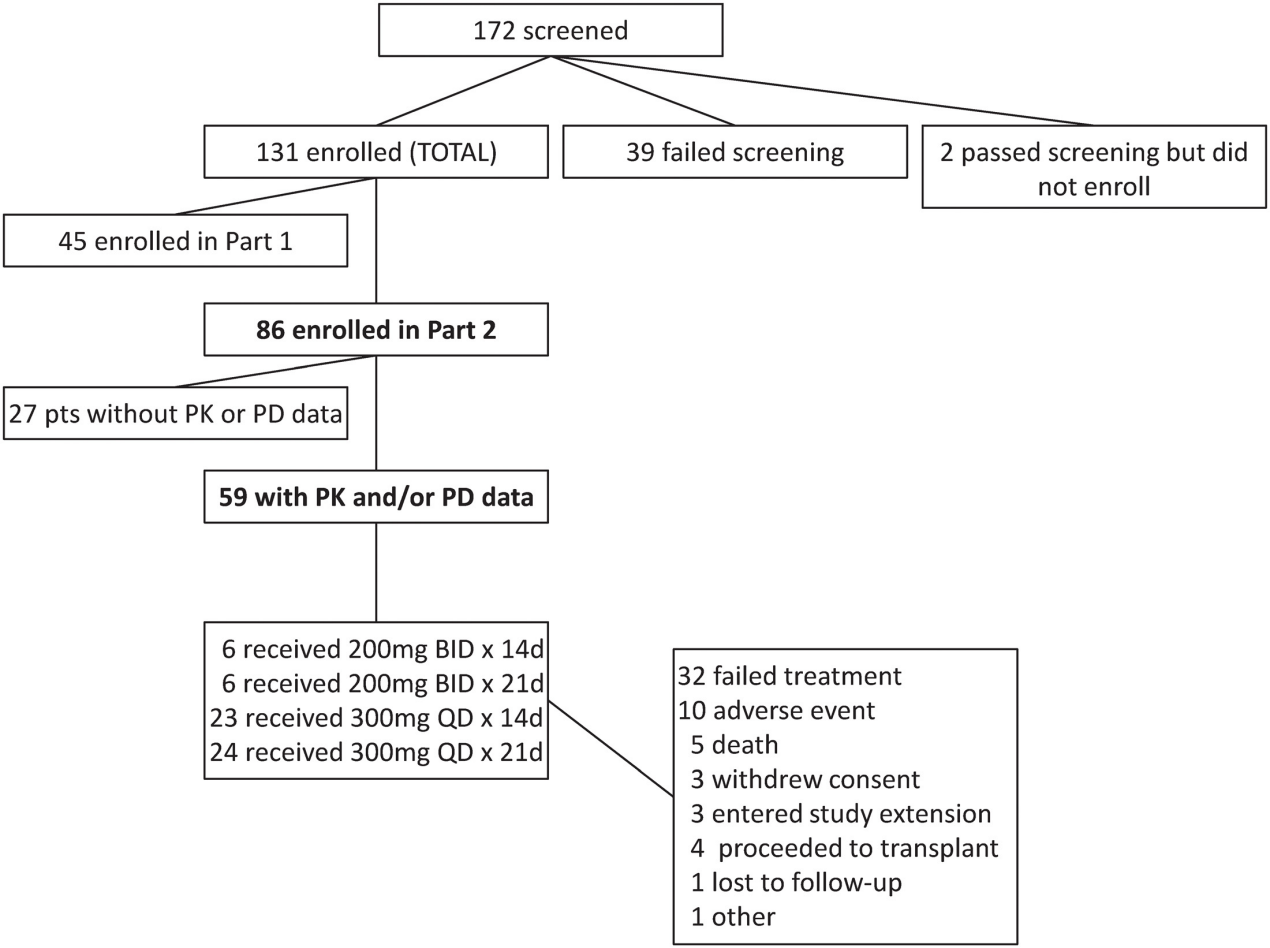
Study flow-chart.

**Fig 2:**
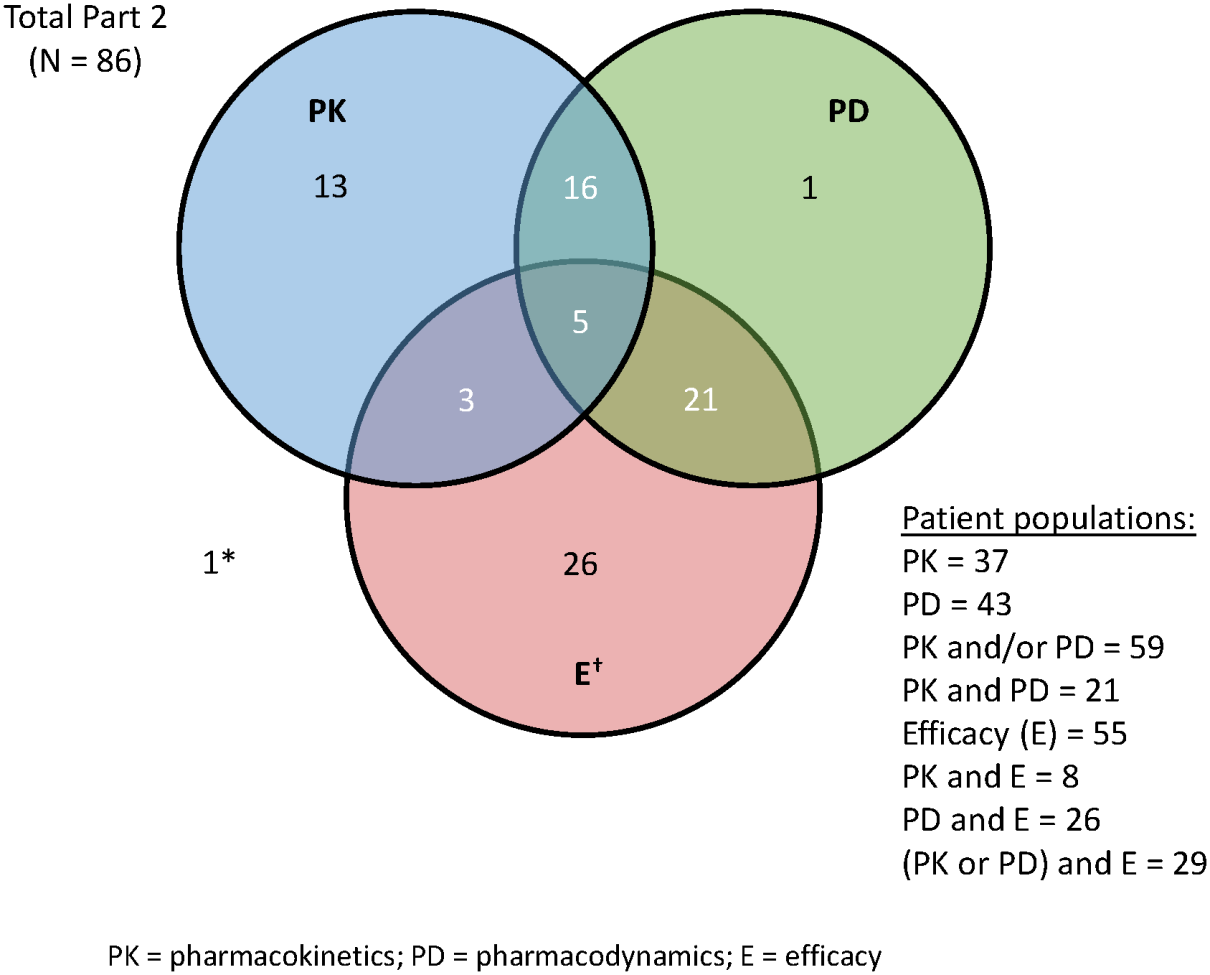
Patient populations.

In all, 86 patients were enrolled in part 2 of this study, 59 of whom had available PK data (n = 37) and/or PD data (n = 43) (Fig 1). Both PK and PD data were available for 21 patients (Fig 2). Baseline demographics and disease characteristics of these 59 patients are shown in Table 1. Numbers of patients in individual dosing groups were: 300 mg once daily x 14 days (n = 23), 300 mg once daily x 21 days (n = 24), 200 mg twice-daily x 14 days (n = 6), and 200 mg twice-daily x 21 days (n = 6).

**Table 1:** Baseline demographics of patients with available PK and/or PD samples.

	All patients
	(N = 59)
**Age (years), mean [SD]**	67.8 [12.3]
**Male gender, n (%)**	40 (67.8)
**Diagnosis (WHO classification), n (%)**	
AML (N = 15)	
*De novo*	9 (15.3)
Transformed from MDS	6 (10.2)
MDS (N = 41)	
RA/RARS/RCMD	20 (33.9)
RAEB-1	9 (15.3)
RAEB-2	4 (6.8)
MDS-U	5 (8.5)
Del(5q)	1 (1.7)
Unknown	2 (3.4)
CMML (N = 3)	3 (5.1)
**MDS IPSS risk classification, n (%)** *	
Low / Intermediate-1	34 (83.0)
Intermediate-2 / High	7 (17.0)
**MDS cytogenetics, n (%)** *	
Normal/Diploid	19 (46.3)
≥1 abnormality	14 (34.1)
Indeterminate	4 (9.8)
Not done	4 (9.8)

*Numbers and percentages based on MDS patients only

BID = twice-daily; QD = once-daily; AML = acute myeloid leukemia; MDS = myelodysplastic syndromes; RA = refractory anemia; RARS = RA with ringed sideroblasts; RCMD = refractory cytopenias with multilineage dysplasia; RAEB = RA with excess blasts; MDS-U = MDS unclassifiable; Del(5q) = deletion in the long arm of chromosome 5; CMML = chronic myelomonocytic leukemia; IPSS = Intermediate Prognostic Scoring System

### PK results

**Fig 3:**
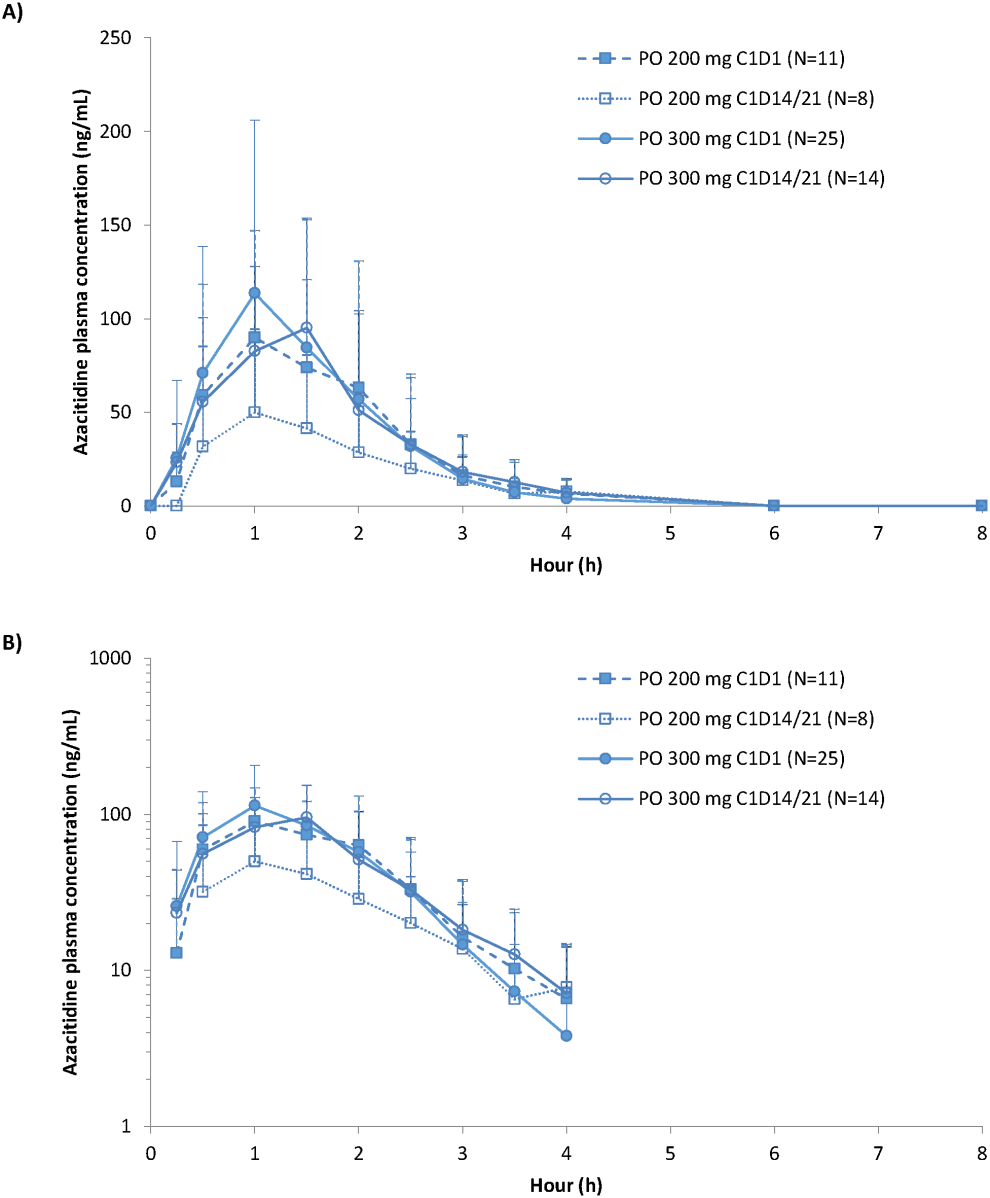
Azacitidine plasma concentrations over time following CC-486 administration (200 mg and 300 mg doses) on the first day (day 1) and last day (day 14/21) of cycle 1. (A) linear and (B) semi-log scales.

PK data were available for 36 patients on day 1 (PK data unavailable for 1 patient) and for 22 patients on the last dosing day (ie, day 14 or 21). Mean azacitidine plasma concentration over time profiles are shown in Fig 3. Relatively high inter-patient variability was noticed for all dose levels and regimens. Overall, dosing profiles were similar in shape across CC-486 treatment schedules, and show that azacitidine was rapidly absorbed, reaching mean C_max_ within 1.50 hours post-ingestion with both the 200 mg and 300 mg doses. Azacitidine concentration then decreased in a multiphasic manner and was not quantifiable from the 6 hour time point onward. There was high inter-patient variability (ie, large percent coefficients of variation [%CV]) observed for azacitidine concentrations (S1 Fig).

Other PK parameters also showed high intra- and inter-patient variability (Table 2). AUC_∞_ and C_max_ values observed on day 1 following 200 mg dosing were similar to values observed on day 1 and day 14/21 following 300 mg dosing, whereas, as expected, observed values with the 200 mg dose on day 14/21 were approximately 50% less than the values observed for the 300 mg dose. Tissue distribution was extensive, as indicated by large apparent volume of distribution (Vz/F). Mean [±SD] CC-486 plasma t_1/2_ ranged from 0.53 [0.17] to 0.78 [0.26] hours across treatment arms. No drug accumulation was noted following multiple-dose administration.

**Table 2:** CC-486 PK parameters on day 1 and the last dosing day (day 14 or 21) of cycle 1.

		AUC_inf_	C_max_	T_max_ ^a^	t_1/2_	Cl/F	Vz/F
		(ng*h/mL)	(ng/mL)	(h)	(h)	(L/h)	(L)
**CC-486 200 mg BID**							
Mean ± SD	Day 1	184 ± 118	121 ± 89		0.56 ± 0.17	1648 ± 1200	1369 ± 1306
(%CV)	(n = 11)	(65)	(73)		(31)	(73)	(95)
Median [min, max]		148 [45, 443]	101 [18, 339]	1.48 [0.50, 3.00]	0.48 [0.36, 0.87]	1353 [452, 4443]	774 [452, 4916]
	Day 14/21	108 ± 79	59 ± 55		0.78 ± 0.26^b^	2827 ± 1699^b^	3501 ± 2623^b^
	(n = 8)	(73)^b^	(94)		(33)	(60)	(75)
		61 [38, 231]	27 [18, 170]	1.25 [0.57, 4.00]	0.67 [0.44, 1.15]	3286 [866, 5208]	4107 [836, 7379]
**CC-486 300 mg QD**							
Mean ± SD	Day 1	193 ± 139	124 ± 84.9		0.53 ± 0.17	2391 ± 2062	1791 ± 1407
(%CV)	(n = 25)	(72)	(69)		(32)	(86)	(79)
Median [min, max]		154 [28, 687]	92 [24, 388]	1.00 [0.47, 2.00]	0.46 [0.34, 0.98]	1948 [437, 10817]	1332 [355, 6202]
	Day 14/21	182 ± 102^c^	98 ± 53		0.62 ± 0.20^c^	2221 ± 1435^c^	2218 ± 2254^c^
	(n = 14)	(56)	(54)		(32)	(65)	(102)
		135 [47, 419]	75 [24, 206]	1.23 [0.50, 3.50]	0.57 [0.41, 0.99]	2225 [716, 6427]	1423 [539, 9142]

%CV = coefficient of variation; AUC_∞_ = the area under the concentration-time curve (AUC) from the time of dosing extrapolated to infinity; CL/F = apparent total clearance; C_max_ = maximum observed concentration; t_1/2_ = terminal half-life; T_max_ = time to maximum concentration; Vz/F = apparent volume of distribution.

^a^Median [min, max] only.

^b^n = 7

^c^n = 13

### PD results

**Fig 4:**
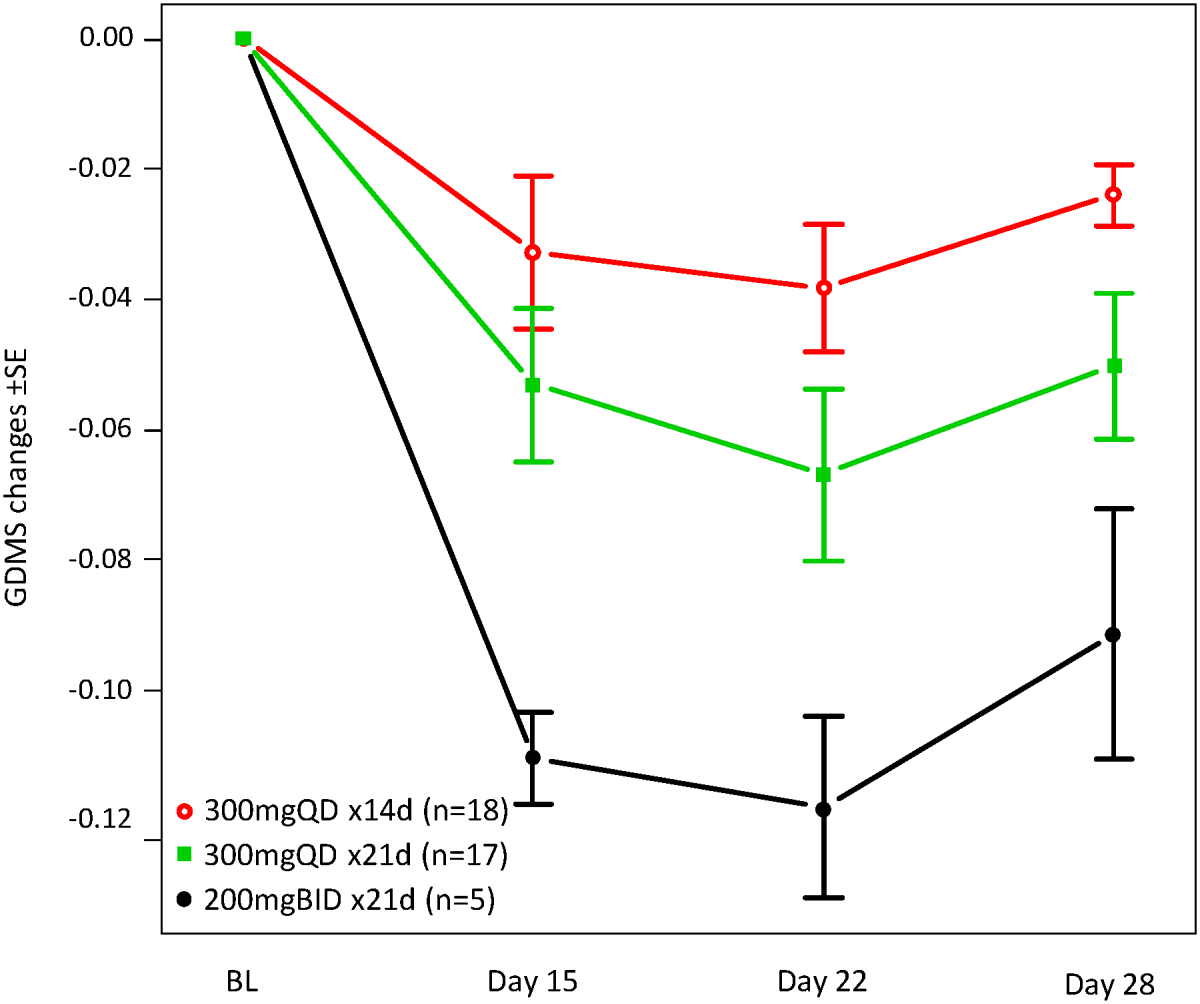
Mean GDMS changes in cycle 1 following CC-486 administration in extended dosing schedules. * (*Too few samples were available to evaluate GDMS changes over time with the CC = 486 200 mg twice daily x 14-day dosing regimen).

Global DNA methylation across 26,486 autosomal loci was reported as the GDMS for each whole blood sample collected throughout the first cycle of therapy; data were available for 35 patients in the total 300 mg once-daily dosing schedule group (14-day n = 18, 21-day n = 17) and for 8 patients in the total 200 mg twice-daily dosing group (14-day n = 3, 21-day n = 5). The extent and significance of GDMS changes between time points were assessed by Wilcoxon signed-rank test (Table 3 and Fig 4). GDMS with 300 mg once daily dosing was significantly reduced with both 14- and 21-day schedules at all measured time points: median differences at day 15 (-1.6% and -4.5%, respectively), day 22 (-2.8% and -6.0%) and cycle end (-2.3% and -4.9%). Greater methylation reduction was observed at cycle end (day 28) with the CC-486 300 mg once-daily 21-day dosing schedule than with the 14-day dosing schedule (-0.38% vs. -1.4%; *P* = .03). The largest absolute reductions in DNA methylation were observed with the 200 mg twice-daily 21-day regimen; however, there were a small number of patients in this group.

**Table 3:** Changes in global DNA methylation score (GDMS) with CC-486 in extended dosing schedules.

	Changes in GDMS							
	Day 15 vs. Baseline		Day 22 vs. Baseline		Cycle End/Day 28 vs. Baseline		Day 22 vs. Day 15	
	median % difference	*P* value*	median % difference	*P* value*	median % difference	*P* value*	median % difference	*P* value*
**CC-486 300 mg QD x 14d (n = 18)**	-1.6%	0.0039	-2.8%	<0.001	-2.3%	<0.001	-0.38%	0.25
**CC-486 300 mg QD x 21d (n = 17)**	-4.5%	<0.001	-6.0%	<0.001	-4.9%	0.0029	-1.4%	0.0084
**CC-486 200 mg BID x 14d (n = 3)**	-5.9%	0.5	+2.6%	N/A^†^	-4.6%	0.5	N/A^‡^	N/A
**CC-486 200 mg BID x 21d (N = 5)**	-11%	0.063	-12.4%	0.13	-7.7%	0.25	-1.2%	0.25

*Wilcoxon signed-rank *P* value

^†^n = 1

^‡^Of the 3 patients in the 200 mg BID x14d group, 2 had an available PD sample only on day 15, and the third had a PD sample only on day 22, preventing the day 15 vs. 22 comparison.

n = the number of patients in a specific subject population; the actual sample size for each comparison may vary slightly due to missing data at various time points.

BID = twice-daily; QD = once-daily

**Fig 5:**
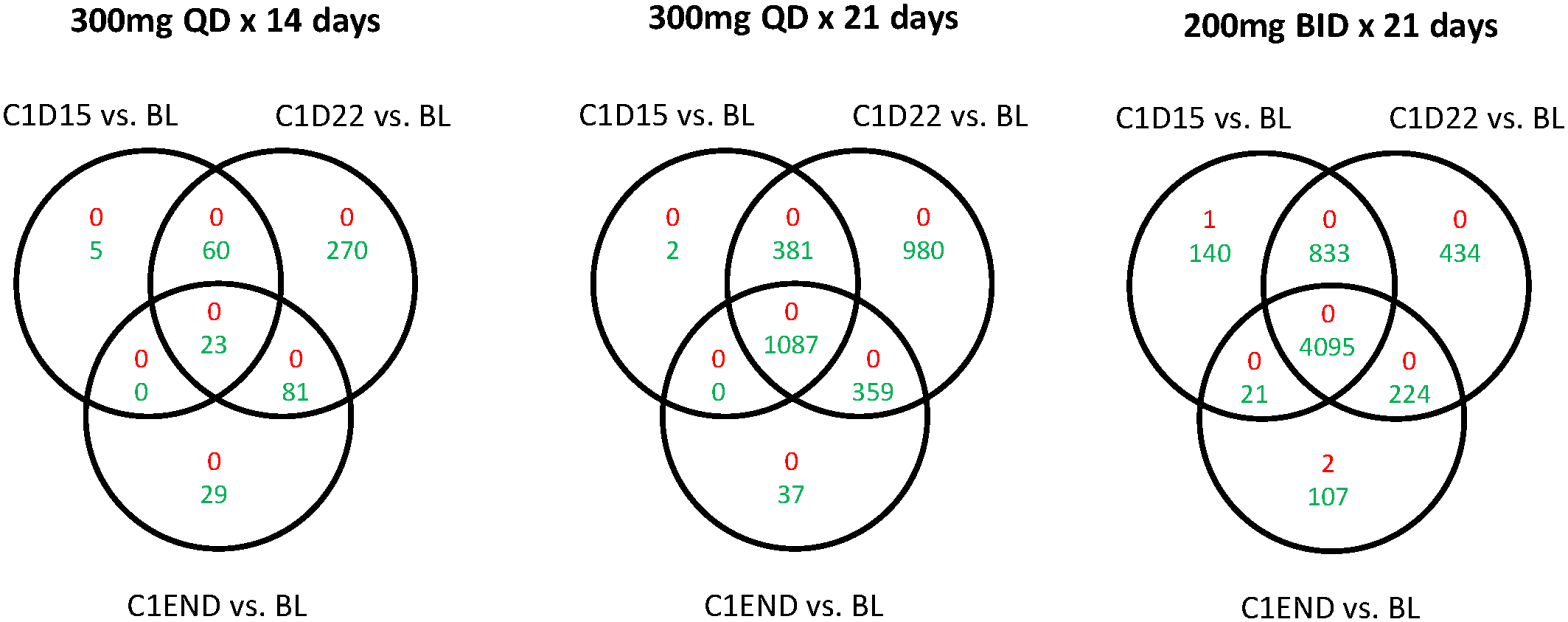
Venn diagrams showing overlaps of significantly regulated genomic loci, by CC-486 dosing schedule. Red numbers represent upregulated loci; green numbers represent down-regulated loci.

The numbers of individual loci with methylation changes at each time point for each dosing regimen are shown in Table 4. The overlap of hypomethylated loci across regimens/times is shown in Fig 5. The greatest demethylation effect was observed on day 22 across treatment groups (Tables 3 and 4), with the exception of the 200 mg twice-daily 14-day schedule, in which no significantly hypomethylated loci were observed, potentially due to small sample size (n = 3). The number of loci with significantly reduced methylation at cycle end was greatest for the 200 mg twice-daily 21-day schedule, followed by the 300 mg once-daily 21-day and 14-day schedules, consistent with what was observed for GDMS changes. The extent and significance of local DNA methylation changes were also evaluated at select genes (S2 Table and S2 Fig). Methylation of CDKN2B and CDH1, genes previously reported for tracking the PD activity of hypomethylating agents, [33, 34] showed wide variation and predominantly low baseline methylation levels (averaged baseline methylation levels of all 10 CDKN2B loci and 6 of 8 CDH1 loci across patients were below 0.5), making them poor surrogates for the global hypomethylating effect of azacitidine. CDH1 locus cg24765079 was an exception, with significant methylation changes observed at all time-points post-treatment with both once-daily dosing schedules and with the 200 mg twice-daily 21-day schedule. Methylation changes in immune checkpoint genes (PD1/PDCD1, PDL1/CD274, PDL2/PDCD1LG2, and CTLA4) were also detected. The kinetics of methylation change in the top 5 CpG loci with the greatest hypomethylation following 21-dosing of CC-486 300 mg once daily (listed in S3 Table) are shown in S3 Fig; methylation changes in these 5 loci reflected global changes, as measured by GDMS (Fig 4).

**Table 4:** Numbers of significantly regulated genomic loci, by CC-486 dosing schedule.*

	Cycle 1—Day 15		Cycle 1—Day 22		Cycle 1 End (Day 28±1)	
Cycle 1	Up	Down	Up	Down	Up	Down
**CC-486 300 mg QD x 14 days (n = 18)**	0	88	0	434	0	133
**CC-486 300 mg QD x 21 days (n = 17)**	0	1470	0	2807	0	1483
**CC-486 200 mg BID x 21 days (n = 5)**	1	5089	0	5586	2	4447

* No loci were significant at the 0.05 level for the CC-486 200 mg BID x14 days dosing regimen, mainly due to small sample size (n = 3).

BID = twice daily; QD = once daily

Pathways potentially regulated by methylation changes were evaluated by mapping hypomethylated loci at day 22 (the day of maximal methylation change) to specific genes and pathways. With the 300 mg once-daily 21-day dosing schedule, 2,807 loci showed significant hypomethylation at day 22. The promoter-associated loci (2,127/2,807) mapped to 2,404 genes (S3 Table), which were significantly (Fisher’s exact test *P* < .01 and minimum number of overlapping genes = 5) enriched for genes involved in multiple canonical pathways, including cytokine/chemokine signaling and G-protein coupled receptor (GPCR) signaling (Table 5 and S4 Table).

**Table 5:** Top 20 biological pathways affected by CC-486 treatment (ranked in order of significance, assessed by Fisher’s exact test. Additional significant pathways are shown in S4 Table.

Pathway
Cytokine-cytokine receptor interaction
Genes involved in Class A/1 (Rhodopsin-like receptors)
Genes involved in GPCR ligand binding
Genes involved in Peptide ligand-binding receptors
Genes involved in Transmembrane transport of small molecules
Genes involved in G alpha (i) signaling events
Complement and coagulation cascades
Hematopoietic cell lineage
Chemokine signaling pathway
Genes involved in Biological oxidations
Genes involved in Chemokine receptors bind chemokines
Neuroactive ligand-receptor interaction
Genes involved in Hemostasis
Genes involved in Metabolism of lipids and lipoproteins
Genes involved in SLC-mediated transmembrane transport
Jak-STAT signaling pathway
Genes involved in Innate Immune System
Steroid hormone biosynthesis
Genes involved in Complement cascade
Genes involved in G alpha (q) signaling events

### PK/PD correlation

**Fig 6:**
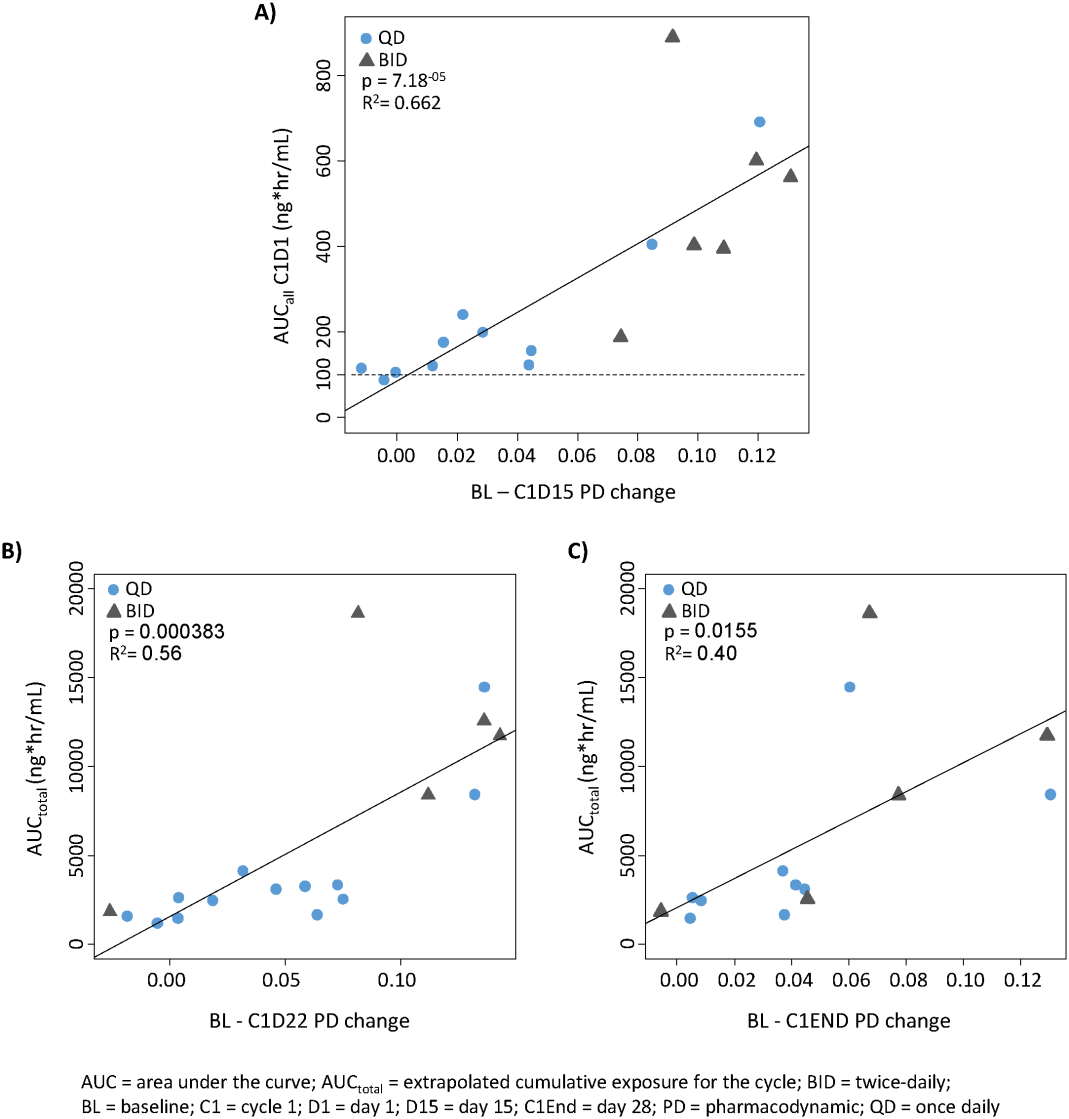
Correlation between azacitidine exposures and methylation changes. **(A)** Azacitidine AUC at Cycle 1, Day 1 (ng*hr/mL) vs methylation change at Cycle 1, Day 15; **(B)** Extrapolated cumulative azacitidine AUC for Cycle 1 vs methylation change at Cycle 1, Day 22, and **(C)** vs methylation change at Cycle 1, Day 28 (cycle end).

A significant PK/PD correlation (AUC on day 1 of cycle 1 vs. change in GDMS between baseline and day 15 of cycle 1) was observed (r^2^ = 0.662; *P* = .0000712), with a minimum biologically effective plasma exposure of approximately 100 ng*hr/mL (Fig 6A). Cumulative drug exposure (AUC_total_) over treatment cycle 1 was also significantly correlated with methylation changes at day 22 and day 28 (cycle end) (Fig 6B and 6C). Cumulative drug exposures may be more clinically relevant than dose levels in exposure–PD analyses (Fig 6B and 6C).

### Efficacy/PD correlation

**Fig 7:**
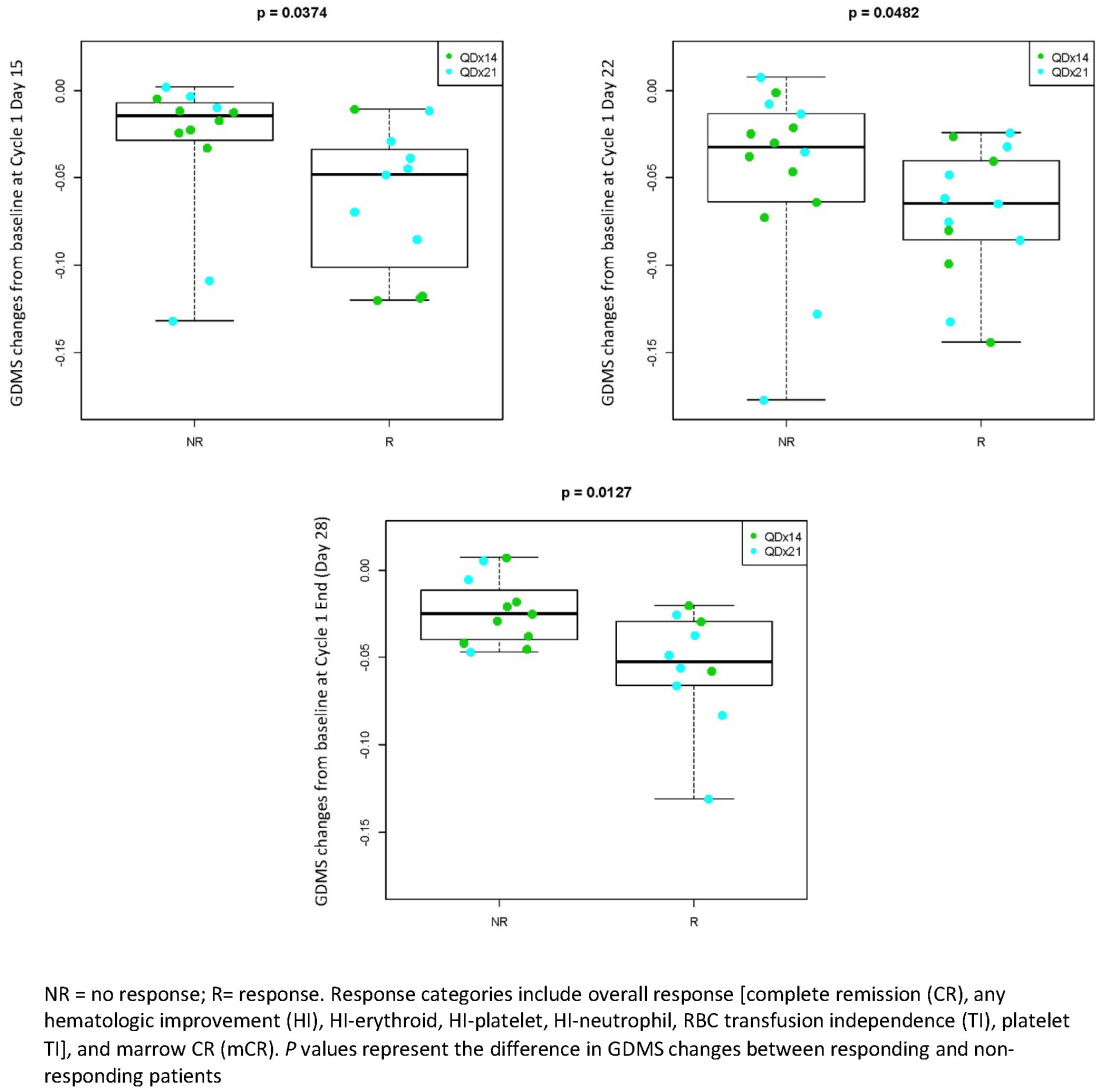
Changes in GDMS during cycle 1 by clinical response (any CC-486 treatment cycle).

**Fig 8:**
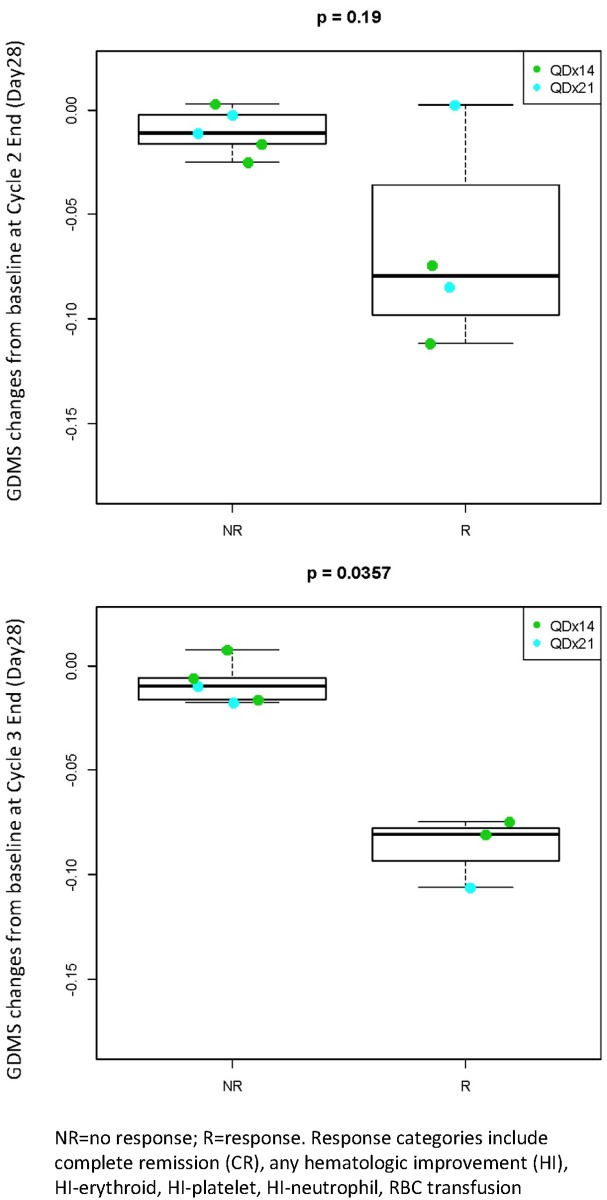
Changes in GDMS at cycle 2 end and cycle 3 end by clinical response (any CC-486 treatment cycle). NR = no response; R = response. Response categories include complete remission (CR), any hematologic improvement (HI), HI-erythroid, HI-platelet, HI-neutrophil, RBC transfusion.

Patients with a clinical response to CC-486 treatment had significantly greater reductions in methylation than non-responding patients at all times during cycle 1 (Fig 7). Similar trends were observed in patients profiled at the end of cycles 2 and 3 (Fig 8).

## Discussion

The reversible nature of epigenetic modifications provides an opportunity for therapeutic targeting in diseases driven by epigenetic aberrancies, but also a challenge in that the demethylating effects of epigenetic therapies are transient. In part 1 of this study, evaluation of DNA methylation changes in whole blood as a pharmacodynamic measure of activity of SC azacitidine 75 mg/m^2^ administered for the first 7 days of a 28-day cycle showed a significant, though transient, decrease in DNA methylation that reached nadir mid-cycle, but ultimately reversed in the second half of the treatment cycle [20]. CC-486 dosing over the same schedule (7 days per 28-day cycle) in part 1 showed similar kinetics of methylation change, with a lesser extent of DNA hypomethylation [20]. However, extending CC-486 dosing to 14 days and 21 days led to sustained significant methylation reductions until the end of the 28-day cycle, potentially preventing aberrant remethylation of CpG islands in genes implicated in hematologic malignancies.

Data from this study show a correlation between global methylation reduction and therapeutic response to CC-486. Compared with SC azacitidine dosing, [20] lower daily exposure achieved with oral dosing over an extended period may prove to be a more effective therapeutic regimen. There is some evidence that lowering the dosage of a hypomethylating agent (which should decrease cytotoxic effects) and administering the lower dose over a longer period (which should increase S-phase-dependent DNA incorporation) may enhance efficacy [35]. Smaller studies of parenteral azacitidine to evaluate the relationship between treatment-induced methylation reduction and response have shown no correlation or mixed results [7, 36]. However, these studies investigated methylation changes at only 2 to 4 gene loci, chosen for their purported role in MDS and AML. In contrast, the current study evaluated global demethylation over more than 27,000 loci spanning 14,495 genes, which may have increased the likelihood of detecting this correlation. The observed association between clinical response and extent of methylation change lends support to the purported epigenetic mechanism of azacitidine; however, observed changes in methylation could represent a surrogate effect of other azacitidine-mediated (eg, RNA-mediated) PD effects and changes in cell populations due to epigenetic and/or non-epigenetic mechanisms. Given the number of patients with PK or PD *and* efficacy data, further research is needed to elucidate relationships among CC-486 exposure, methylation changes, and clinical response.

Consistent with results of part 1 of this study [20] and of other studies, [37, 38] high inter- and intra-patient variability was observed for azacitidine concentrations and PK parameters. The reasons for this variability are unknown. Azacitidine metabolism is driven by the enzyme, cytidine deaminase (CDA) [38]. CDA is highly polymorphic, [39] which may help explain inter-individual differences in azacitidine PK. Moreover, serum CDA levels can vary within an individual subject for several reasons, including physical exercise, [40] perhaps leading to intra-subject variations in PK. However, these ideas are speculative. Extending the number of days of azacitidine administration by two to three times that of 7-day SC azacitidine dosing did not alter PK. Due to the short plasma half-life of azacitidine, daily CC-486 dosing over 14 or 21 days showed no evidence of drug accumulation, nor was there evidence of decreased absorption after multiple doses. Azacitidine plasma exposure (AUC) and methylation reductions were significantly correlated (*P* < .0000712) as evidenced by the fact that decreases in highly methylated loci were greatest in the 200 mg twice-daily 21-day dosing schedule.

Previous studies have shown a relatively consistent pattern in the kinetics of change in DNA methylation levels induced by SC azacitidine [7, 41, 42] and for CC-486 administered for 7 days in part 1 of this study [20]. SC azacitidine and CC-486 in part 1 were associated with methylation reductions of highly methylated loci that were maximal at approximately 15 days after treatment initiation [20]. The current analysis shows that extending CC-486 dosing for 14 and 21 days per 28-day cycle led to maximum hypomethylation at 22 days, with persistent DNA hypomethylation at the end of the treatment cycle. Pathway analysis of regulated loci shows that multiple pathways are affected, as would be expected based on the global hypomethylating mechanism of azacitidine. The immunomodulatory effects of azacitidine *in vivo* implicate significant enrichment of regulated genes involved in cytokine/chemokine signaling pathways [43, 44]. Interestingly, observed methylation changes in immune checkpoint genes (S3 Fig) are consistent with a recent report of upregulated expression of these genes in patients with MDS treated with epigenetic therapy, [45] and provide a rationale for combining CC-486 with checkpoint inhibitor therapies.

Oral administration of azacitidine could provide a number of potential benefits, including maximizing convenience to patients, preventing injection- and catheter-site reactions, eliminating the need for clinic accessibility on weekends, reducing resource costs associated with frequent clinic visits, improving adherence to therapy, and providing flexibility for extended dosing schedules and maintenance therapy. Two phase 3 trials of extended CC-486 dosing schedules (*NCT01757535* and Clinicaltrials.gov *NCT01566695*) have been initiated to evaluate CC-486 given for 14 days/cycle as maintenance therapy after complete remission in patients with AML, and for 21 days/cycle to assess clinical efficacy and safety in patients with lower-risk MDS.

## Supporting Information

S1 Fig (A) AUC∞ and (B) C_max_ values for individual patients during the first CC-486 treatment cycle. (DOC)

S2 Fig Line plots of DNA methylation changes over time by dosing regimen at the loci shown in S2 Table in individual patients. (DOC)

S3 Fig Mean changes in methylation levels over the entire treatment cycle with three CC-486 treatment schedules for the top 5 most significantly hypomethylated loci on day 22 with the 300mg QDx21day regimen (*see* S3 Table). The error bars correspond to ± 1 standard error.
(DOC)

S1 Protocol Trial protocol. (PDF)

S1 Table Institutional Review Boards. (DOCX)

S2 Table DNA methylation changes at select genes. (DOC)

S3 Table Hypomethylated loci on day 22 for the CC-486 300 mg once-daily 21-day dosing schedule. Kinetics of mean methylation changes in the top 5 hypomethylated loci are shown in S3 Fig.
(DOC)

S4 Table Overlap of significantly enriched genes involved in multiple biological pathways affected by CC-486 treatment. (XLSX)

S1 TREND Checklist Trend checklist. (PDF)
