# Supplementary material for: Pharmacokinetics and Pharmacodynamics with Extended Dosing of CC-486 in Patients with Hematologic Malignancies
Source: PLoS One. 2015 Aug 21;10(8):e0135520. doi: 10.1371/journal.pone.0135520 (PMC4546409; doi:10.1371/journal.pone.0135520)
Supplement: S2 Fig — (DOC) [file pone.0135520.s002.doc]

**Supplementary Figure 2.**
