# Supplementary material for: Pharmacokinetics and Pharmacodynamics with Extended Dosing of CC-486 in Patients with Hematologic Malignancies
Source: PLoS One. 2015 Aug 21;10(8):e0135520. doi: 10.1371/journal.pone.0135520 (PMC4546409; doi:10.1371/journal.pone.0135520)
Supplement: S3 Fig — The error bars correspond to ± 1 standard error. (DOC) [file pone.0135520.s003.doc]

**Supplementary Figure 3**. Changes in methylation levels of the 5 most significantly hypomethylated loci by the 21-day CC-486 300 mg once daily regimen on day 21 (cg19949550 for ASB2, cg04968473 for CYP1A2, cg04797496 for PCDH12, cg07826255 for SGCA, and cg09022808 for GAL3ST1; *see Supplementary Table 3*) across 3 treatment cohorts. The mean profiles across patients within each cohort are plotted and the error bars correspond to +/- 1 standard error.
