# Supplementary material for: Pharmacokinetics and Pharmacodynamics with Extended Dosing of CC-486 in Patients with Hematologic Malignancies
Source: PLoS One. 2015 Aug 21;10(8):e0135520. doi: 10.1371/journal.pone.0135520 (PMC4546409; doi:10.1371/journal.pone.0135520)
Supplement: S1 Protocol — (PDF) [file pone.0135520.s004.pdf]

PROTOCOL AMENDMENT 6

A PHASE 1, OPEN-LABEL, DOSE-ESCALATION STUDY TO EVALUATE THE SAFETY, PHARMACOKINETICS, AND PHARMACODYNAMICS OF ORAL AZACITIDINE IN SUBJECTS WITH MYELODYSPLASTIC SYNDROMES (MDS), CHRONIC MYELOMONOCYTIC LEUKEMIA (CMML) OR ACUTE MYELOGENOUS LEUKEMIA (AML)

**INVESTIGATIONAL PRODUCT (IP):**	**Oral Azacitidine (CC-486)**
**PROTOCOL NUMBER:**	**AZA PH US 2007 CL 005**
**DATE ORIGINAL PROTOCOL FINAL:**	**02 May 2007**
**DATE AMENTMENT 6 FINAL**	**10 Jul 2015**

**EudraCT Number:**	**Not Applicable**
**IND NUMBER:**	**074618**

TABLE OF CONTENTS

TITLE PAGE 1
1. STUDY OBJECTIVES 5
1.1. Primary Objectives 5
1.2. Secondary Objectives 5
2. STUDY DESIGN 6
2.1. Overall Study Design 6
2.1.1. Part 1 6
2.1.2. Part 2 7
2.1.3. Optional Extension Phase 8
2.2. Dose Escalation 8
2.2.1. Part 1 8
2.2.2. Part 2 9
2.2.3. Expanded Evaluation 11
2.2.4. Treatment Duration 11
2.2.5. Treatment Assignment and Intrasubject Dose Escalation 12
2.2.6. Treatment Continuation 12
2.3. Dose-Limiting Toxicity 12
2.4. MTD Expansion 13
2.5. Study Duration and Dates 14
2.6. Pharmacokinetics 14
2.7. Correlative Studies (Pharmacodynamics) 15
3. SELECTION OF SUBJECTS 16
3.1. Number of Subjects 16
3.2. Inclusion Criteria 16
3.3. Exclusion Criteria 17
3.4. Inclusion and Exclusion Criteria for OEP 17
3.5. Subjects or Partners of Subjects of Reproductive Potential 18
3.6. Waivers of Inclusion/Exclusion Criteria 18
4. STUDY TREATMENTS 19
4.1. Treatments to be Administered 19
4.1.1. Subcutaneous Azacitidine Drug Supply 20

4.1.2. Oral Azacitidine Drug Supply 20
4.2. Treatment Administration and Schedule 20
4.2.1. Dietary Restrictions for Subjects in Part 1 20
4.2.2. Dietary Restrictions for Subjects in Part 2 20
4.2.3. Dietary Restrictions for Subjects in OEP 21
4.2.4. Subcutaneous Azacitidine Dispensing and Administration....21
4.2.5. Oral Azacitidine Dispensing and Administration....21
4.3. Dose Modifications....23
4.3.1. Dose Modifications....23
4.3.2. Dose Modifications Based on Renal Function and Serum Electrolytes....23
4.3.3. Dose Modifications Based on Diarrhea....24
4.3.4. Retreatment Criteria....24
4.3.5. Intrasubject Dose Escalation....25
4.4. Method of Treatment Assignment....25
4.4.1. Part 1 25
4.4.2. Part 2 25
4.4.3. OEP....25
4.5. Blinding, Packaging, and Labeling....25
4.6. Supplies and Accountability....25
4.7. Compliance....26
5. STUDY PROCEDURES....27
5.1. Screening Procedures....27
5.2. On-Study Evaluations....28
5.2.1. On-Study Evaluations for Subjects in Part 1 29
5.2.2. On-Study Evaluations for Subjects in Part 2 40
5.2.3. Optional Extension Phase....48
5.3. End of Study/Subject Discontinuation....48
5.4. Additional Follow-Up....49
5.5. Optional Extension Phase....49
6. DATA MEASUREMENTS AND METHODS OF COLLECTION 50
6.1. Data Measurements 50
6.1.1. Safety Data....50
6.1.2. Pharmacokinetic Data....50

6.1.3. Correlative Studies Data (Pharmacodynamic).....52
6.1.4. Efficacy Data.....52
6.2. Methods of Collection.....52
6.2.1. Pharmacokinetic Sample Collection.....52
6.2.2. Sample Collection for Correlative Studies (Pharmacodynamics).....54
6.2.3. Safety Measurements.....56
7. DISCONTINUATIONS.....59
7.1. Discontinuation of Subjects.....59
7.2. Replacement of Subjects.....59
7.3. Study Stopping Rules.....60
8. STATISTICAL METHODS.....61
8.1. Determination of Sample Size.....61
8.2. Analysis Populations.....61
8.2.1. Safety Population.....61
8.2.2. Dose-Limiting Toxicity Evaluable Population.....61
8.2.3. Pharmacokinetic Population.....62
8.2.4. Pharmacodynamic Population.....62
8.2.5. Efficacy Evaluable Population.....62
8.3. Analysis Methods.....62
8.3.1. Safety.....62
8.3.2. Pharmacokinetics.....63
8.3.3. Correlative Studies (Pharmacodynamics).....64
8.3.4. Efficacy.....64
8.4. Analysis from OEP.....64
9. REFERENCES.....65

1. STUDY OBJECTIVES

1.1. Primary Objectives

The primary objectives are:

- To determine the MTD of oral azacitidine on different treatment schedules;
- To determine dose-limiting toxicities (DLTs) of oral azacitidine;
- To determine the safety profile of oral azacitidine;
- To evaluate the pharmacokinetic (PK) behavior of azacitidine administered orally and SC; and
- To evaluate the pharmacodynamic (PD) effects of azacitidine administered orally and SC.

1.2. Secondary Objectives

The secondary objectives are:

- To evaluate hematological response and/or improvement rate according to revised IWG criteria; and
- To determine the biologically active dose based on safety, PK, and PD data.
- For the optional extension phase: to evaluate long term safety of oral azacitidine.

2. STUDY DESIGN

2.1. Overall Study Design

This is a multicenter, open-label, Phase 1, sequential design, dose-escalation study of oral azacitidine. The study is designed to evaluate the MTD, DLTs, safety, PK profiles, and PD profiles of increasing doses of orally administered azacitidine on different treatment schedules.

The study will be conducted in 2 parts with an optional extension phase. Part 1 is designed to evaluate oral azacitidine on a 7-day QD treatment schedule and evaluate the plasma PK and PD of azacitidine administered orally in comparison to those of azacitidine administered SC at the approved dose of 75 mg/m^2/day. Part 2 is designed to evaluate oral azacitidine on 14-day QD, 14-day BID, 21-day QD and 21-day BID treatment schedules. Part 2 will begin once the MTD in Part 1 has been determined and the MTD expansion cohort for that schedule has fully enrolled.

The OEP allows subjects continuing to receive oral azacitidine and who have stable disease or are demonstrating clinical benefit as assessed by the Investigator, to receive oral azacitidine in an OEP until they meet the criteria for study discontinuation (Section **Error! Reference source not found.**) or oral azacitidine becomes commercially available. Details for the OEP are provided in Appendix M, Section **Error! Reference source not found.**.

2.1.1. Part 1

Subjects will be enrolled into the study in cohorts of 3. During Cycle 1, all subjects will receive SC azacitidine at the approved dose of 75 mg/m^2/day for the first 7 days of the 28-day cycle. The PK and PD profile of SC administered azacitidine (75 mg/m^2/day) will be evaluated during Cycle 1. During Cycle 2, each subject will receive oral azacitidine once daily for the first 7 days of the 28-day cycle. Subjects will be evaluated for the MTD, DLTs, safety, PK, and PD profiles during Cycle 2. Subjects can continue to receive oral azacitidine for Cycles 3 and beyond. Subjects continuing to receive oral azacitidine will have additional assessments of safety, PK, and PD performed during Cycles 3 and beyond. Subjects confirmed to be non-responders after 6 cycles of oral azacitidine (Cycle 7 on study) may discontinue from the study protocol and study treatment, or remain in the study and cross over to receive SC azacitidine at the approved dose of 75 mg/m^2/day for the first 7 days of each 28-day cycle, or continue with oral azacitidine treatment (possibly at a higher dose). Subjects will only be allowed to cross over to SC azacitidine after completion of Cycle 7 prior to starting Cycle 8.

2.1.1.1. Cycle 1: SC Azacitidine

Cohorts of 3 subjects will be entered into the study and will receive azacitidine administered by SC injection at the approved dose of 75 mg/m^2/day for the first 7 days of Cycle 1, regardless of the oral azacitidine dose they are scheduled to receive. During Cycle 1, PK and PD data will be generated for SC azacitidine in all cohorts.

2.1.1.2. Cycle 2 and Beyond: Oral Azacitidine

2.1.1.2.1. Starting and Stopping Dose Rationale

Based on data from the pilot oral dosing study with azacitidine (AZA PH US 2006 PK004 protocol), a starting dose of 120 mg/day (approximately 67 mg/m^2) for the first 7 days of each 28-day cycle (starting with Cycle 2) was chosen for this study. After one cohort has completed the assessments for the first oral azacitidine cycle, if appropriate, the dose will be escalated by 60 mg (to 180 mg) for the next cohort as described in Section 2.2, Dose Escalation. Following evaluation of the 360 mg dose, dose escalation will occur in increments of 120 mg, until the MTD is reached.

2.1.2. Part 2

Subjects will be enrolled into the study in cohorts of 6. During Cycles 1 and beyond, subjects will receive oral azacitidine QD or BID for the first 14 or 21 days of each 28-day cycle. Subjects will be evaluated for the MTD, DLTs, safety, PK, and PD profiles during Cycle 1. Subjects can continue to receive oral azacitidine for Cycles 2 and beyond. Subjects continuing to receive oral azacitidine will have additional assessments of safety, PK, and PD performed during Cycles 2 and beyond. Subjects confirmed to be non-responders after 6 cycles of oral azacitidine (Cycle 6 on study) may discontinue from the study protocol and study treatment, or remain in the study and cross over to receive SC azacitidine at the approved dose of 75 mg/m^2/day for the first 7 days of each 28-day cycle, or continue with oral azacitidine treatment (possibly at a higher dose). Subjects will only be allowed to cross over to SC azacitidine after completion of Cycle 6 prior to starting Cycle 7.

2.1.2.1. Treatment Schedules

Four different treatment schedules are planned for evaluation in Part 2 of the study:

- QD, Days 1 – 14;
- BID, Days 1 – 14;
- QD, Days 1 – 21; and
- BID, Days 1 – 21.

The 14-day QD treatment schedule will be evaluated first, followed by dose escalation on that schedule and/or simultaneous or subsequent evaluation of one or more of the other treatment schedules. The decision to proceed with dose-escalation on the 14-day QD treatment schedule and/or enrollment into one or more of the other treatment schedules will be made based on safety observed in the initial 14-day QD cohort treated with 300 mg oral azacitidine, and will be agreed upon between the Investigators and the Sponsor's Medical Monitor. Treatment schedules may be evaluated sequentially or concurrently as agreed upon between the Investigators and Sponsor's Medical Monitor.

2.1.2.2. Starting Dose Rationale

The starting dose for the 14-day QD treatment schedule, which will be evaluated first, will be 300 mg. The starting dose in the 14-day BID, 21-day QD, and 21-day BID treatment schedules

is anticipated to be 100 mg, 200 mg, or 300 mg. The actual starting dose in each treatment schedule will be determined by safety observed in previously evaluated cohorts, and will be agreed upon between the Investigators and the Sponsor's Medical Monitor prior to subject enrollment in that treatment schedule.

2.1.3. Optional Extension Phase

Details for the Study Design of the OEP are provided in Appendix M, Section **Error! Reference source not found.**.

2.2. Dose Escalation

The dose escalation phase in Part 1 of the study will utilize a standard "3 + 3" design to estimate the MTD for azacitidine administered orally. A description of the statistical characteristics (probabilities of halting or continuing dose escalation) of this design is provided in Appendix K, Section **Error! Reference source not found.**. The dose escalation phase in Part 2 of the study will utilize a design that evaluates 6 subjects per cohort to estimate the MTD for azacitidine administered orally. A description of the statistical characteristics (probabilities of halting or continuing dose escalation) of this design is provided in Appendix L, Section **Error! Reference source not found.**. MTD will be defined as the highest dose at which no more than 33% of the subjects observed at a given dose level in a treatment schedule experience a DLT.

2.2.1. Part 1

Subjects will be individually assessed for safety and DLT in the first cycle of oral azacitidine treatment (Cycle 2). Three eligible subjects will be enrolled in sequential cohorts at increasing dose levels until at least 1 DLT is seen during the first cycle of oral azacitidine treatment (Cycle 2). The starting dose of orally administered azacitidine will be 120 mg administered daily for 7 consecutive days. The following dose escalation rules will be used and applied to first cycle of oral azacitidine therapy (Cycle 2):

1. Three subjects are initially studied at each dose level.
2. If none of these 3 subjects experience DLT, then the dose is escalated in increments of 60 mg (up to 360 mg) or 120 mg (above 360 mg) in 3 subsequent subjects.
3. If 1 of these 3 subjects experiences DLT at the current dose, then up to 3 more subjects are accrued at the same level.
   a. If none of these 3 additional subjects experience DLT, then the dose is escalated in increments of 60 mg (up to 360 mg) or 120 mg (above 360 mg) in subsequent subjects after the last subject in the lower cohort completes 1 cycle on oral azacitidine treatment.
   b. If 1 of these 3 additional subjects experiences DLT, then subject entry at that dose level is stopped, and that dose level will be considered the MTD.
   c. If 2 or more of these 3 additional subjects experiences DLT, then the MTD is considered to have been exceeded and the previous dose level will be the MTD.
4. If 2 or more of the initial 3 subjects at a dose level experience DLT, then the MTD is considered to have been exceeded and dose escalation will be stopped. Up to 3 more subjects will be treated at the next lower dose. In the event that the MTD is determined

to have been exceeded at the starting dose of 120 mg, then a dose of 60 mg will be evaluated in 3 additional subjects.

A subject must meet one of the following 3 criteria to be evaluable for dose-escalation decisions:

- received at least 5 of the 7 scheduled doses and has completed Cycle 2, Day 28 without a DLT;
- experienced a DLT; or
- withdrawn from the study prior to completing Cycle 2, Day 28 due to a DLT.

If a subject does not meet any of these criteria, the subject is not evaluable for dose escalation decisions and will be replaced in that cohort. No more than 3 subjects will be added simultaneously to a dose cohort during dose escalation.

Figure 1 provides an example of how the MTD will be determined during Cycle 2.

Figure 1: Example of DLT Flow Diagram for Part 1

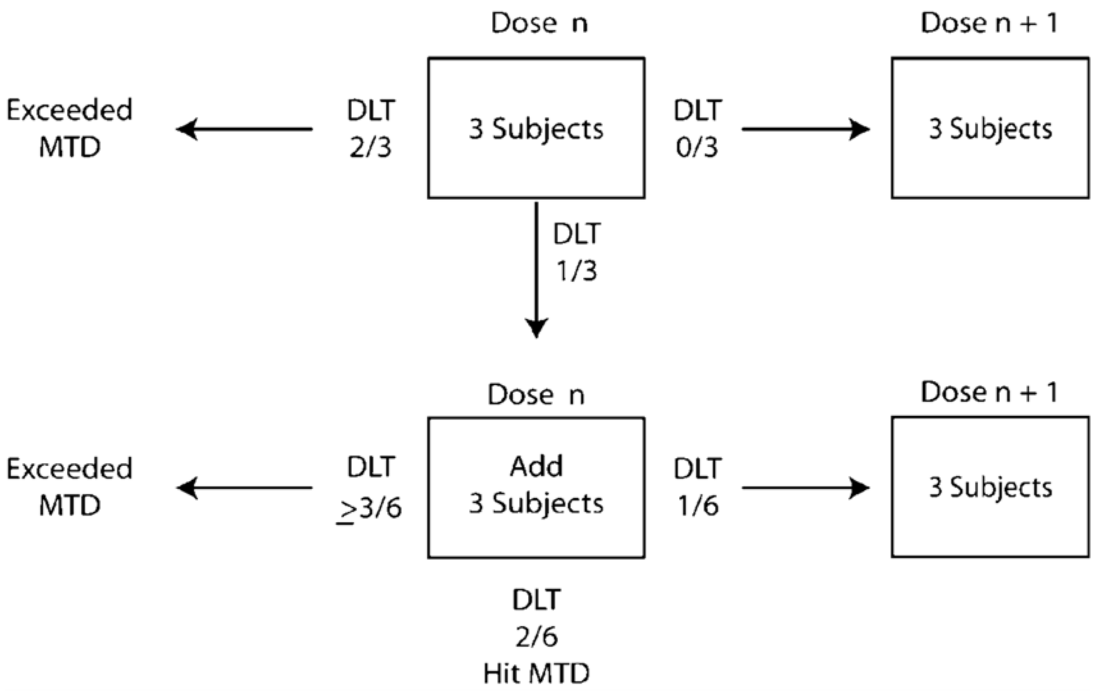

2.2.2. Part 2

Subjects will be enrolled in cohorts of 6. During Cycles 1 and beyond, subjects will receive oral azacitidine QD or BID for the first 14 or 21 days of each 28-day cycle. Subjects will be individually assessed for safety and DLT in the first cycle of oral azacitidine treatment (Cycle 1). Six eligible subjects will be enrolled in sequential cohorts at increasing dose levels in a treatment schedule until at least 2 DLTs are observed during the first cycle of oral azacitidine treatment (Cycle 1). MTD is defined as the highest dose at which no more than 33% of the subjects observed at a given dose level in a treatment schedule experience a DLT. The following dose escalation rules will be used and applied to the first cycle of oral azacitidine therapy (Cycle 1):

1. Six subjects are initially studied at each dose level.

2. If ≤ 1 of these 6 subjects experience DLT, then the dose is escalated in increments of 100 mg in 6 subsequent subjects.
3. If 2 of these 6 subjects experience DLT, then subject entry at that dose level is stopped, and that dose level will be considered the MTD.
4. If ≥ 3 of these 6 subjects experience DLT, then the MTD is considered to have been exceeded and dose escalation will be stopped and the next lower dose level will be considered the MTD. In the event that the MTD is determined to have been exceeded at the starting dose, then the dose will be reduced by 100 mg and another 6 subjects evaluated.

A subject must meet one of the following criteria to be evaluable for dose-escalation decisions:

- received at least 10 of 14 scheduled doses in the 14-day QD treatment schedule, or at least 20 of 28 scheduled doses in the 14-day BID treatment schedule, or at least 15 of 21 scheduled doses in the 21-day QD treatment schedule, or at least 30 of 42 scheduled doses in the 21-day BID treatment schedule in Cycle 1, and has completed Cycle 1, Day 28 without a DLT; or
- experienced a DLT in Cycle 1.

If a subject does not meet either of these criteria, the subject is not evaluable for dose escalation decisions and will be replaced in that cohort. No more than 6 subjects will be added simultaneously to a dose cohort during dose escalation.

Figure 2 provides an example of how the MTD will be determined for the different treatment schedules in Part 2.

Figure 2: Example of DLT Flow Diagram for Part 2

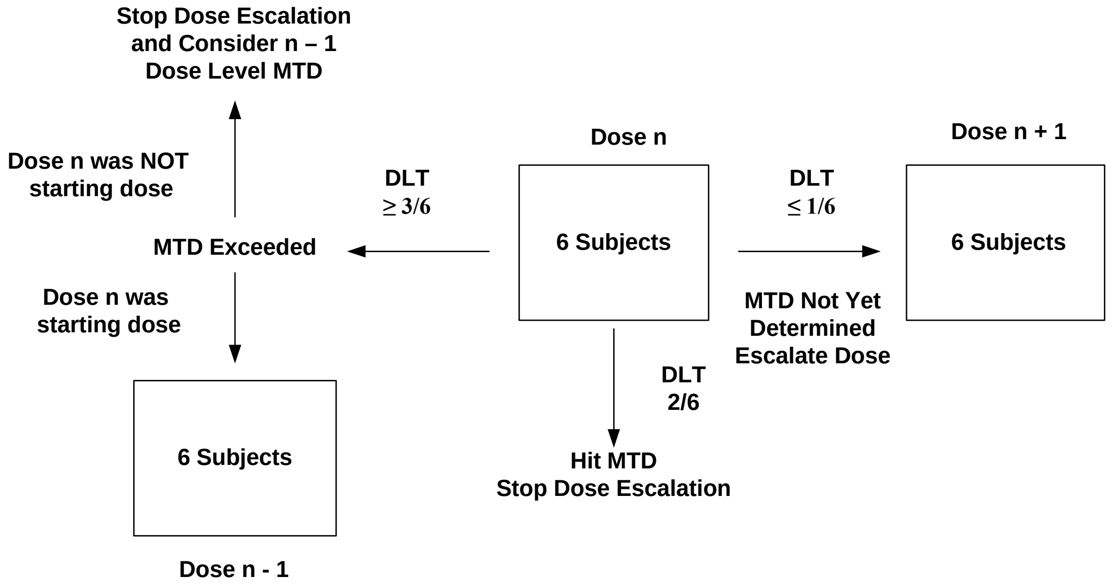

2.2.3. Expanded Evaluation

Additional subjects may be treated at any previously evaluated dose level in a treatment schedule in order to better characterize safety and PK/PD parameters. Intermediate or lower dose levels may also be explored following discussion and agreement between the Investigators and the Sponsor's Medical Monitor. Institutional Review Boards will be notified accordingly if intermediate or lower dose levels are investigated. Prior to initiating each new dose level or expanding an existing dose level, a safety teleconference will be scheduled wherein Investigators and the Sponsor's Medical Monitor will confer and document agreement that such a step is considered appropriately safe. To establish a recommended dose and schedule for evaluation in future efficacy studies, a minimum of 20 subjects should be treated.

In the event that MTD is not reached on one or more treatment schedules and/or the Investigators and Sponsor's Medical Monitor agree that further dose escalation on one or more treatment schedules is not warranted, approximately 20 low and/or Int-1 risk MDS subjects, not inclusive of those treated prior to implementation of Amendment #4, may be enrolled and treated at a previously evaluated dose on a given treatment schedule and/or at a lower dose than previously evaluated on that schedule. Expanded treatment schedules may be evaluated concurrently or sequentially as agreed upon between the Investigators and Sponsor's Medical Monitor.

To reduce the risk of exposing subjects to an excessively toxic dose level, a stopping rule will be implemented if more than 33% of subjects in a treatment schedule expansion arm experience a DLT. For example, if after 12 subjects are enrolled and treated 5 are found to have experienced a DLT then enrollment into that treatment schedule expansion arm will stop. At least 12 subjects should be enrolled into a treatment schedule expansion arm before this rule will take effect.

2.2.4. Treatment Duration

Because responses to azacitidine may require 4 to 6 cycles of therapy, subjects will not be removed from study due to documentation of progressive disease on interim bone marrow investigations obtained in response to hematologic toxicity provided that subject performance status has not significantly deteriorated and that continued administration of study therapy is not deemed detrimental to the subject's health. Subjects may continue to receive additional cycles of oral azacitidine treatment beyond Cycle 7 (subjects in Part 1) or Cycle 6 (subjects in Part 2), provided they do not have disease progression as defined by revised IWG criteria for hematologic response and/or improvement (see Appendix D, Section **Error! Reference source not found.**; Appendix I, Section **Error! Reference source not found.**; and Appendix J, Section **Error! Reference source not found.**), clinically significant proliferative disease that requires additional cytotoxic chemotherapy, or a clinically significant drug-related adverse event (AE) that does not resolve or respond to treatment intervention after 3 weeks.

Subjects enrolled in the OEP may continue to receive oral azacitidine until they meet the criteria for study discontinuation (Section **Error! Reference source not found.**) or until the availability of a rollover protocol exists, into which any subjects who remain on study may be consented and continue to receive treatment with oral azacitidine. It must be the opinion of the investigator that the remaining subject(s) continue to receive benefit from treatment with oral azacitidine. Details for the OEP are provided in Appendix M, Section **Error! Reference source not found.**.

2.2.5. Treatment Assignment and Intrasubject Dose Escalation

All subjects will remain on the treatment schedule to which they are assigned, unless the treatment schedule is deemed to not be safe, in which case reassignment to another schedule will be agreed upon between the Investigator and the Sponsor's Medical Monitor. Intrasubject dose escalation of oral azacitidine will be permitted once a subject completes 2 cycles (subjects in Part 1) or 4 cycles (subjects in Part 2) of oral therapy without experiencing a significant drug-related toxicity, provided the dose level to which the subject will escalate has already been evaluated and has a DLT rate ≤ 33% at the time of the dose adjustment. The decision to escalate a subject's dose will be made between the Sponsor's Medical Monitor and Investigator prior to any discussion with the subject. There are no limits to the number of dose escalations a subject may have, provided the above considerations are met. The dose may be increased at the beginning of the cycle and cannot be changed during the 7, 14 or 21 days of treatment.

Refer to Section **Error! Reference source not found.** for Treatment Assignment in the OEP.

2.2.6. Treatment Continuation

Subjects will continue or discontinue therapy after 6 cycles of treatment with oral azacitidine according to the following criteria:

1. Subjects responding to oral azacitidine treatment (see Appendix D, Section **Error! Reference source not found.**; Appendix I, Section **Error! Reference source not found.**; and Appendix J, Section **Error! Reference source not found.**) for revised IWG hematologic response/improvement criteria may:
   a. Continue with oral azacitidine treatment according to the protocol; or
   b. Discontinue the study protocol and study treatment.
2. Subjects not responding to oral azacitidine may:
   a. Discontinue the study protocol and study treatment;
   b. Cross over to SC azacitidine treatment; or
   c. Continue with oral azacitidine treatment (possibly at a higher oral dose, only if a higher dose has been evaluated positively).

Upon a site's IRB approval of protocol Amendment 5 which allows for an OEP, any subject enrolled in Part 1 or Part 2 continuing to receive oral azacitidine who has stable disease or is demonstrating clinical benefit as assessed by the Investigator, and has consented to participate in Amendment 5, may enter the OEP of this study (at their current dose) at the start of their next cycle.

Subjects may continue to receive oral azacitidine in the OEP until they meet the criteria for study discontinuation (Section **Error! Reference source not found.**) or oral azacitidine becomes commercially available. Details for the OEP are provided in Appendix M, Section **Error! Reference source not found.**.

2.3. Dose-Limiting Toxicity

DLT will be defined as any of the following events that are determined by the Investigator to be related to treatment with oral azacitidine and will constitute a change from baseline irrespective of outcome.

1. Grade 3 or greater nausea, diarrhea, or vomiting despite the use of adequate/maximal medical intervention;
3. Any other clinically significant nonhematologic toxicity of grade 3 or greater considered not related to the underlying disease or intercurrent illness;
4. Any treatment-related effect leading to a subject missing:
   a. 3 or more doses of oral azacitidine in a 7-day QD treatment schedule;
   b. 5 or more doses of oral azacitidine in a 14-day QD treatment schedule;
   c. 9 or more doses of oral azacitidine in a 14-day BID treatment schedule;
   d. 7 or more doses of oral azacitidine in a 21-day QD treatment schedule; or
   e. 13 or more doses of oral azacitidine in a 21-day BID schedule;
5. Failure of recovery to an absolute neutrophil count (ANC) > 500/µL and/or platelets > 25,000/µL with a hypocellular marrow (< 5%) by 42 days after the start of a subject's first cycle of oral azacitidine. Subjects with a baseline ANC ≤ 500/µL and/or platelets ≤ 25,000/µL will not be evaluable for neutrophil or platelet toxicity. The time course and degree of neutrophil and/or thrombocytopenia for these subjects will be monitored and reviewed during regularly scheduled safety teleconferences between the Investigators and the Sponsor's Medical Monitor; or
6. Any nonhematologic toxic effect considered to be related to oral azacitidine and resulting in the delay of a subject's second cycle of oral azacitidine by > 14 days.

For the purpose of dose-escalation decisions and MTD determination, only DLTs that occur during the first cycle of oral azacitidine treatment will be taken into account. If any subject experiences a DLT while completing a later cycle of therapy, a review of these data will be included in the regularly scheduled safety teleconferences between the Investigators and the Sponsor's Medical Monitor.

2.4. MTD Expansion

Once the MTD has been determined for a treatment schedule, an expanded evaluation of safety, PK, PD, and hematologic response and/or improvement rate at this dose level may be conducted in additional subjects so that the total number of subjects exposed to this dose level in that treatment schedule is up to 47 subjects. The exposure of additional subjects at the MTD will provide a better estimate of the toxicity rate.

To reduce the risk of exposing subjects to an excessively toxic dose level, a stopping rule will be implemented if more than 33% of subjects in an expansion arm experience a DLT. For example, if after 12 subjects are enrolled and treated (inclusive of those treated during the dose escalation phase of the study), 5 are found to have experienced a DLT then enrollment into that MTD expansion arm will stop. At least 12 subjects should be enrolled into a MTD expansion arm before this rule will take effect.

Should an MTD expansion arm in a particular treatment schedule be terminated prematurely due to safety concerns, a lower dose level and/or different treatment schedule may be evaluated.

2.5. Study Duration and Dates

The subject recruitment period is approximately 48 months, starting third quarter 2007. The study duration encompasses the following:

Part 1:

- Screening period of up to 21 days;
- Treatment with subcutaneous (SC) azacitidine for Cycle 1. Cycle 1 will be a 28-day cycle;
- Treatment with oral azacitidine for Cycles 2 and beyond in 28-day cycles with a suggested 6-cycle minimum (Cycle 7 on study);
- Crossover to SC azacitidine is permitted, at the discretion of the Investigator, after completion of all Cycle 7 assessments and starting on Day 1 of Cycle 8 for subjects confirmed to be non-responders to oral azacitidine; and
- 28-day follow-up.

Part 2:

- Screening period of up to 21 days;
- Treatment with oral azacitidine for Cycles 1 and beyond in 28-day cycles with a suggested 6-cycle minimum (Cycle 6 on study);
- Crossover to SC azacitidine is permitted, at the discretion of the Investigator, after completion of all Cycle 6 assessments and starting on Day 1 of Cycle 7 for subjects confirmed to be non-responders to oral azacitidine; and
- 28-day follow-up.

OEP:

Upon a site's IRB approval of protocol Amendment 5 which allows for an OEP, any subject enrolled in Part 1 or Part 2 continuing to receive oral azacitidine who has stable disease or is demonstrating clinical benefit as assessed by the Investigator, and has consented to participate in Amendment 5, may enter the OEP of this study (at their current dose) at the start of their next cycle.

Subjects may continue to receive oral azacitidine in the OEP until they meet the criteria for study discontinuation (Section **Error! Reference source not found.**) or until the availability of a rollover protocol exists, into which any subjects who remain on study may be consented and continue to receive treatment with oral azacitidine. It must be the opinion of the investigator that the remaining subject(s) continue to receive benefit from treatment with oral azacitidine. Details for the OEP are provided in Appendix M, Section **Error! Reference source not found.**.

2.6. Pharmacokinetics

Serial blood samples will be collected following SC and/or oral azacitidine administration to determine plasma azacitidine concentrations. Urine samples will also be collected from subjects

in Part 1 for the determination of azacitidine concentrations. In Part 1, the plasma and urine PK parameters calculated following oral administration in Cycles 2 and 7 will be compared to those obtained following the SC administration in Cycle 1. Additionally, the potential relationship between azacitidine PK and PD profiles and/or responses will be investigated. Please refer to **Error! Reference source not found.**, **Error! Reference source not found.**, **Error! Reference source not found.**, and Section 6.2 of this protocol, and to the Study Reference and Laboratory Manuals, for detailed information on PK sample collection, handling, and shipping procedures for this study.

Note: All PK sampling for Part 2 subjects will be discontinued with implementation of Amendment #4. All Part 1 subjects have completed PK sampling.

2.7. Correlative Studies (Pharmacodynamics)

Pharmacodynamic assessments will be performed on bone marrow aspirate and whole blood samples obtained from each subject following SC and/or oral azacitidine administration.

Measurements of DNA methylation in the DNA extracted from peripheral blood and bone marrow will be used as pharmacodynamic markers of azacitidine activity. DNA methylation measurements may be performed using global and gene-specific DNA methylation assays. Samples for DNA methylation measurements will be collected before exposure to azacitidine (screening/baseline specimen collected no more than 7 days prior to Cycle 1) and after azacitidine administration (see **Error! Reference source not found.**, **Error! Reference source not found.**, **Error! Reference source not found.**, and **Error! Reference source not found.** for detailed collection schedule).

Additional markers that may be used to explore the pharmacodynamic activity of azacitidine in bone marrow include, but are not limited to, DNA methyltransferase protein levels, markers of DNA damage (e.g., gamma-histone H2AX), and gene expression. Additional markers that may be used to explore the pharmacodynamic activity of azacitidine in peripheral blood include gene expression and levels of soluble proteins.

Possible relationships between pharmacodynamic variables, PK parameters and clinical outcomes may be explored if appropriate.

3. SELECTION OF SUBJECTS

3.1. Number of Subjects

It is anticipated that up to 150 subjects may be enrolled in this study at 4 to 10 investigative sites. Justification for the sample size is provided in Section 8.1.

3.2. Inclusion Criteria

Subjects must meet the following criteria in order to be enrolled in the study:

1. Male or female ≥ 18 years of age;
2. Diagnosis of low or Int-1 risk MDS according to IPSS (see Appendix A, Section **Error! Reference source not found.** and Appendix F, Section **Error! Reference source not found.**).
3. Platelet count ≤ 50 x 109/L (≤ 50,000/μL), and/or hemoglobin ≤ 9.0 g/dL, and/or RBC transfusion-dependent and/or platelet transfusion-dependent (see Appendix D, Section **Error! Reference source not found.**);
4. Eastern Cooperative Oncology Group (ECOG) performance status 0 to 2 (see Appendix E, Section **Error! Reference source not found.**);
5. Women of childbearing potential may participate, providing they meet the following conditions (also see Section 3.5):
6. Must agree to use at least 2 physician-approved contraceptive methods throughout the study and for 3 months following the last day of azacitidine dosing; and
7. Must have a negative serum pregnancy test obtained during screening within 7 days prior to azacitidine dosing on Cycle 1, Day 1;
8. Males with female partners of childbearing potential must agree to use at least 2 physician-approved contraceptive methods throughout the study and should avoid fathering a child for 3 months following the last day of azacitidine dosing (also see Section 3.5);
9. Serum creatinine levels ≤ 2.5 x upper limit of normal (ULN);
10. Serum glutamic oxaloacetic transaminase (SGOT [AST]) and serum glutamic pyruvic transaminase (SGPT [ALT]) levels ≤ 2.5 x ULN;
11. Serum bilirubin levels ≤ 1.5 x ULN. Higher levels are acceptable if these can be attributed to active hemolysis (as indicated by positive direct Coomb's test, decreased haptoglobin, Gilbert's disease, elevated indirect bilirubin and/or lactate dehydrogenase [LDH]) or ineffective erythropoiesis (as indicated by bone marrow findings);
12. Serum bicarbonate ≥ 20 mEq/L; and
13. Written informed consent, willingness, and ability to comply with all study procedures.

3.3. Exclusion Criteria

Subjects meeting any of the following criteria will not be included in the study:

1. A diagnosis of acute promyelocytic leukemia;
2. Previous or concurrent malignancy (other than low or Int-1 risk MDS) except adequately treated basal cell or squamous cell skin cancer; in situ carcinoma of the cervix, or other solid tumor treated curatively, and without evidence of recurrence for at least 3 years prior to study entry;
3. Prior treatment with azacitidine or other demethylating agents;
4. Active, uncontrolled clinically significant infections;
5. Presence of serious illness, medical condition, or other medical history, including abnormal laboratory parameters, which, in the opinion of the Investigator, would be likely to interfere with a subject's participation in the study or with the interpretation of the results;
6. Any known or suspected hypersensitivity to azacitidine or mannitol or any other ingredient used in the manufacture of azacitidine drug product (see the Investigator's Brochure);
7. Presence of gastrointestinal disease or other conditions known to interfere with the absorption, distribution, metabolism, or excretion of drugs;
8. Known Human Immunodeficiency Virus (HIV) or Hepatitis C, or known active viral Hepatitis B;
9. Breastfeeding or pregnant females;
10. Treatment with any anticancer therapy (standard or investigational) within the previous 21 days prior to the first dose of study drug or less than full recovery (CTCAE grade 1) from the clinically significant toxic effects of that treatment. The use of hydroxyurea in subjects with rapidly proliferating disease is allowed only during Cycle 1 after SC administration of azacitidine for 7 days for subjects in Part 1 only. Hydroxyurea may be used for two weeks after dosing in Cycle 1 (eg, Days 8-21 dosed with hydroxyurea) for subjects in Part 1 only;
11. Treatment with any investigational drugs within the previous 21 days prior to Cycle 1, Day 1, or ongoing adverse events from previous treatment with investigational drugs, regardless of the time period; or
12. Current congestive heart failure (NY Heart Association Class III-IV), unstable angina or angina requiring surgical or medical intervention within 6 months prior to Cycle 1, Day 1, myocardial infarct within 6 months prior to Cycle 1, Day 1, or uncontrolled cardiac arrhythmia (defined as arrhythmia that is symptomatic or requires treatment or asymptomatic sustained ventricular tachycardia). Subjects with controlled atrial fibrillation that is asymptomatic are eligible.

3.4. Inclusion and Exclusion Criteria for OEP

Refer to Appendix M, Section **Error! Reference source not found.** - Optional Extension Phase.

3.5. Subjects or Partners of Subjects of Reproductive Potential

Female subjects who are more than 2 years postmenopausal or have had a hysterectomy will not be considered of childbearing potential. Women of childbearing potential must use at least 2 physician-approved contraceptive methods while enrolled in the study and for 3 months after the last date of dosing. Adequate forms of contraception are double-barrier methods (eg, condoms with spermicidal jelly or foam and diaphragm with spermicidal jelly or foam), oral, depot, or injectable contraceptives, intrauterine devices, and tubal ligation.

Males with female partners of childbearing potential must agree to use at least 2 physician-approved contraceptive methods throughout the study and should avoid fathering a child for 3 months following the last date of dosing with study drug.

Female subjects of childbearing potential will have pregnancy tests throughout the study. A serum pregnancy is required at screening, within 7 days prior to Cycle 1 Day 1, and at the end of study visit. Additional pregnancy tests (urine or serum) are required within 7 days prior to Day 1 of each dosing cycle and upon discontinuation of treatment during any phase or end of study visit for females of childbearing potential (FCBP) only.

3.6. Waivers of Inclusion/Exclusion Criteria

No waivers of these inclusion or exclusion criteria will be granted by the Investigator and the Sponsor or its designee.

4. STUDY TREATMENTS

All subjects entering the screening phase will receive a unique subject number. This number will be used to identify the subject throughout the study. Subjects withdrawn from the study retain their subject number. New subjects must always be allotted a new subject number.

4.1. Treatments to be Administered

During Cycle 1, all subjects in Part 1 will receive 75 mg/m^2/day azacitidine administered as a SC injection daily for the first 7 consecutive days of the cycle. Cycle 1 will be a 28-day cycle. All doses of SC azacitidine will be administered by study personnel or designee.

During Cycle 2, all subjects in Part 1 will receive oral azacitidine administered at a fixed daily oral dose for the first 7 days of each 28-day cycle. The starting dose of oral azacitidine will be 120 mg/day beginning on Cycle 2, Day 1. Doses of oral azacitidine initially will be escalated in increments of 60 mg. Following evaluation of the 360 mg dose, dose escalation will occur in increments of 120 mg, until the MTD is reached. Intermediate dose increments may also be evaluated. Intrasubject dose escalation of oral azacitidine will be permitted as detailed in Section 4.3.5

Subjects in Part 2 will receive treatment with oral azacitidine QD or BID for the first 14 or 21 days of each 28-day cycle. Doses administered BID will be taken 12 hours (± 30 minutes) apart. The starting dose in the 14-day QD treatment schedule will be 300 mg. The starting dose in the 14-day BID, 21-day QD, and 21-day BID treatment schedules is anticipated to be 100 mg, 200 mg, or 300 mg. The actual starting dose in each of these treatment schedules will be determined based on safety observed in previously evaluated cohorts, and will be agreed upon between the Investigators and the Sponsor's Medical Monitor prior to subject enrollment in that treatment schedule. Doses of oral azacitidine will be escalated in increments of 100 mg until the MTD is reached in that treatment schedule. Intrasubject dose escalation of oral azacitidine will be permitted as detailed in Section 4.3.5.

To ensure subject safety in the dose-escalation portion of the study, an early stopping rule will be implemented in the event that a DLT rate of greater than 33% is observed at the lowest dose level evaluated in a treatment schedule. In the event a DLT rate of greater than 33% is observed at the starting dose in a treatment schedule, then the dose will be reduced and additional subjects evaluated.

Subjects confirmed to be non-responders according to the revised IWG criteria after 6 cycles of treatment with oral azacitidine are allowed to remain on study and continue with oral azacitidine (possible dose increase) or cross over to receive SC azacitidine at the approved dose of 75 mg/m^2/day for the first 7 days of each 28-day cycle. The Sponsor's Medical Monitor should be notified as soon as possible when a subject is crossed over to SC azacitidine.

Upon a site's IRB approval of protocol Amendment 5 which allows for an OEP, any subject enrolled in Part 1 or Part 2 continuing to receive oral azacitidine who has stable disease or is demonstrating clinical benefit as assessed by the Investigator, and has consented to participate in Amendment 5, may enter the OEP of this study (at their current dose) at the start of their next cycle.

Subjects may continue to receive oral azacitidine in the OEP until they meet the criteria for study discontinuation (Section **Error! Reference source not found.**) or oral azacitidine becomes commercially available. Details for treatment in the OEP are provided in Appendix M, Section **Error! Reference source not found.**.

4.1.1. Subcutaneous Azacitidine Drug Supply

Azacitidine will be supplied as a sterile lyophilized powder containing 100 mg of azacitidine and 100 mg of mannitol per vial.

Unreconstituted vials of azacitidine must be stored at 25ºC (77ºF), excursions permitted to 15°C to 30°C (59°F to 86°F). See Section 4.6 for information on the storage of SC azacitidine study drug.

4.1.2. Oral Azacitidine Drug Supply

Azacitidine will be supplied as 20 mg, 60 mg, and 100 mg tablets and as 100 mg capsules. Each tablet and capsule is formulated using excipients that are generally regarded as safe and are used in marketed drug products. Further details can be found in the azacitidine Investigator's Brochure.

Each tablet or capsule will be sealed in a blister package or pouch or packaged in bulk in high-density polyethylene (HDPE) bottles. Oral azacitidine tablets should be stored at 25ºC (77ºF), excursions permitted to 15°C to 30°C (59°F to 86°F). Oral azacitidine capsules should be stored at 2°C to 8°C (35.6°F to 46.4°F). Capsules should be allowed to warm to room temperature for approximately 30 minutes prior to dispensing. The storage area should be secure and have limited access (see Section 4.6). Shelf-life evaluations of the intact blister packages, pouches, and HDPE bottles are ongoing. Oral azacitidine study drug will be monitored for stability for the duration of the study.

4.2. Treatment Administration and Schedule

4.2.1. Dietary Restrictions for Subjects in Part 1

Prior to and during all cycles, subjects should be advised not to consume grapefruit/grapefruit juice for 3 days prior to Day 1 of the cycle and for 15 days after Day 1 of the cycle. Subjects may have grapefruit/grapefruit juice during Day 16 through Day 25 of the cycle. Subjects should be instructed to limit their coffee intake to one 8 ounce cup of black coffee (no cream or sugar) and to not consume alcohol, tea, chocolate, or cola beverages within 2 hours prior to collection of PK samples. In addition, subjects receiving oral azacitidine will have to fast for a minimum of 2 hours prior to ingesting the oral dose of azacitidine and for a minimum of 2 hours after ingesting oral azacitidine. The subject should drink 8 ounces (240 mL) of room temperature water with each dose. Water and any required medications are permitted during the fasting period.

4.2.2. Dietary Restrictions for Subjects in Part 2

Subjects should be advised not to consume any grapefruit/ grapefruit juice during the study, beginning 3 days prior to Cycle 1, Day 1. Subjects should drink 8 ounces (240 mL) of room temperature water with each dose. Oral azacitidine may be taken on an empty stomach or with

food. If the dose is taken in the morning, subjects may consume their usual breakfast before or after administration. The breakfast meal should not exceed 600 calories; however, the actual calorie count will not need to be measured or recorded. If a meal other than breakfast is consumed, a light meal (not more than 25% of a subject's usual daily calories) may be eaten before or after dose administration.

Subjects on a BID dosing schedule should take the 2 doses of oral azacitidine 12 hours (± 30 minutes) apart.

4.2.3. Dietary Restrictions for Subjects in OEP

Details for dietary restrictions in the OEP are provided in Appendix M, Section **Error! Reference source not found.**.

4.2.4. Subcutaneous Azacitidine Dispensing and Administration

Using aseptic technique, reconstitute each vial of azacitidine containing 100 mg of azacitidine with 4 mL sterile water for injection. Inject the diluent slowly into the vial and vigorously shake or roll the vial until a uniform suspension is achieved. The suspension will be cloudy. The resulting suspension will contain azacitidine 25 mg/mL. Do not filter the suspension after reconstitution. Doing so could remove the active substance.

Preparation for Immediate Administration: The product may be held at room temperature for up to 1 hour but must be administered within 1 hour after reconstitution.

Preparation for Delayed Administration: The reconstituted product may be kept in the vial or drawn into a syringe. The product must be refrigerated immediately and may be held under refrigerated conditions (2ºC to 8ºC, 36ºF to 46ºF) for up to 8 hours. After removal from refrigerated conditions, the suspension may be allowed to equilibrate to room temperature for up to 30 minutes prior to administration.

To provide a homogeneous suspension, the contents of the syringe must be re-suspended by inverting the syringe 2 to 3 times and vigorously rolling the syringe between the palms for 30 seconds immediately prior to administration.

Doses greater than 4 mL should be divided equally into 2 syringes and injected into 2 separate sites. Rotate sites for each injection (thigh, abdomen, or upper arm). New injections should be given at least 1 inch from an old site and never into areas where the site is tender, bruised, red, or hard.

On the first day of dosing, subjects meeting all of the inclusion and none of the exclusion criteria will be administered an SC dose of azacitidine. Dosing can occur at any time during the morning after completing the required predose assessments. The exact time of dosing must be recorded in the source documents.

4.2.5. Oral Azacitidine Dispensing and Administration

4.2.5.1. Part 1

On Cycle 2, Day 1, subjects who meet the retreatment criteria (Section 7) will be administered an oral dose of azacitidine tablets or capsules with 8 ounces (240 mL) of room temperature water. Subjects should fast a minimum of 2 hours prior to ingesting the oral dose of azacitidine and a

minimum of 2 hours after ingesting study drug. Water and any required concomitant medications are permitted during the fasting period. Dosing can occur at any time during the morning after completing the required predose assessments. The exact time of dosing must be recorded in the source documents. Each dose of oral azacitidine should be taken with 8 ounces (240 mL) of room temperature water; the total amount of water ingested with the study drug will be recorded in the CRF and subject source documents for PK sample collection days only.

Subjects will return to the clinic for dosing on Days 3 and 7 during Cycle 2. On Days 2, 4, 5, and 6, subjects will take study drug at home. Subjects should be given a quantity of study drug for the dosing days at home. Subjects will be instructed to inspect each azacitidine tablet or capsule and only take tablets or capsules that are totally intact. Subjects should be instructed to return any tablet or capsule found to be not totally intact to the study clinic. Subjects should be instructed to open the study medication packaging as close as possible to when they are going to take the study drug and record the time of study drug administration in their diary card. On days when study drug is taken at home or on days when PK samples are not collected during the clinic visit, subjects should be encouraged to ingest oral azacitidine on an empty stomach with 8 ounces (240 mL) of room temperature water and refrain from ingesting food for approximately 2 hours after dosing.

During Cycles 3 and beyond (except Cycle 7, detailed below), subjects will return to the clinic for dosing on Day 1 and will take study drug at home on Days 2 through 7. During Cycle 7, subjects will return to the clinic on Day 7 for PK blood samples (see **Error! Reference source not found.**, **Error! Reference source not found.**, and Section 5 for study procedure details).

4.2.5.2. Part 2

In the clinic, on Day 1 of each cycle, subjects will be administered an oral dose of azacitidine tablets or capsules with 8 ounces (240 mL) of room temperature water. Dosing can occur at any time during the morning after completing the required predose assessments. The exact time of dosing must be recorded in the source documents. Each dose of oral azacitidine should be taken with 8 ounces (240 mL) of room temperature water; the total amount of water ingested with the study drug will be recorded in the CRF and subject source documents. Subjects may consume their usual breakfast before or after dose administration. The breakfast meal should not exceed 600 calories; however, the actual calorie count will not need to be measured or recorded. If a meal other than breakfast is consumed, a light meal (not more than 25% of a subject's usual daily calories) may be eaten before or after dose administration.

On Days 2-14 or Days 2-21 of each cycle, depending on the treatment schedule to which a subject is assigned, subjects will take study drug at home or in the clinic. Subjects should be given a quantity of study drug for the dosing days at home. Subjects will be instructed to inspect each azacitidine tablet or capsule and only take tablets or capsules that are totally intact. Subjects should be instructed to return any tablet or capsule found to be not totally intact to the study clinic. Subjects should be instructed to open the study medication packaging as close as possible to when they are going to take the study drug and record the time of study drug administration in their diary card.

During Cycles 2 and beyond (except Cycle 6, detailed below), subjects will return to the clinic for dosing on Day 1 and will take study drug at home or in the clinic on Days 2-14 or Days 2-21.

4.2.5.3. OEP

Refer to Appendix M, Section **Error! Reference source not found.** for Oral Azacitidine Dispensing and Administration in the OEP.

4.3. Dose Modifications

4.3.1. Dose Modifications

4.3.1.1. Subcutaneous Azacitidine

Dose modifications for subjects receiving SC azacitidine should be based on guidelines described in the Vidaza® package insert (see Section **Error! Reference source not found.**).

4.3.1.2. Oral Azacitidine - Part 1 and Part 2

Subjects may skip scheduled doses of oral azacitidine during Cycle 2 (subjects in Part 1) or during Cycle 1 (subjects in Part 2) in the event of toxicity that does not meet DLT criteria, and/or at the discretion of the Investigator.

All subjects who experience significant toxicity possibly or probably related to oral azacitidine should have the dose of the study drug reduced between 60 mg (minimum) and 120 mg (maximum). One additional dose reduction step between 60 mg and 120 mg can be made if drug-related toxicities persist or recur in the subsequent cycle. If toxicities persist or recur after the second dose reduction, study treatments will be terminated. The Sponsor's Medical Monitor must be consulted prior to any dose reduction step.

During the course of the study, the 20 mg and 60 mg dose strength tablets may be discontinued and replaced by 100 mg dose strength tablets or capsules. Each subject enrolled into Part 1 of the study and ongoing at the time the 20 mg and 60 mg dose strength tablets are discontinued will have his or her dose adjusted up or down to the nearest multiple of 100 mg.

The Sponsor's Medical Monitor and the Investigator will confer and agree upon the appropriate dose for each subject in advance of such a dose adjustment.

4.3.1.3. Oral Azacitidine - OEP

Refer to Appendix M, Section **Error! Reference source not found.** for Dose Modifications in the OEP.

4.3.2. Dose Modifications Based on Renal Function and Serum Electrolytes

Part 1 or Part 2:

If unexplained reductions in serum bicarbonate levels to less than 20 mEq/L occur, the dosage should be reduced on the next course. Dose reductions will be between 60 mg (minimum) and 120 mg (maximum). Similarly, if unexplained elevations of blood urea nitrogen (BUN) and/or serum creatinine occur, the next cycle should be delayed until values return to baseline and the dose should be reduced between 60 mg and 120 mg on the next treatment course. One additional dose reduction step can be made if reductions in bicarbonate levels persist or elevations in BUN and/or creatinine recur in the subsequent cycle. If the toxicities persist or recur after the second dose reduction, study treatment will be terminated. If the unexplained reductions in serum

bicarbonate or elevations in BUN and/or creatinine occur during the first cycle of treatment with oral azacitidine, the subject's dose should be reduced.

Should similar unexplained renal and/or electrolyte disturbances subsequently persist or recur during the subject's next cycle of treatment with oral azacitidine, study treatment will be discontinued. The Sponsor's Medical Monitor must be consulted prior to any dose reduction step.

OEP:

Refer to Appendix M, Section **Error! Reference source not found.** for Dose Modifications Based on Renal Function and Serum Electrolytes in the OEP.

4.3.3. Dose Modifications Based on Diarrhea

Oral azacitidine has been associated with diarrhea. It is recommended that subjects experiencing diarrhea be managed according to the guidelines provided in Appendix H, Section **Error! Reference source not found.** of the protocol. A dose reduction of azacitidine may be appropriate based on the severity of the diarrhea observed and the response to treatment intervention. The Sponsor's Medical Monitor must be consulted prior to any dose reduction step.

Refer to Appendix M, Section **Error! Reference source not found.** for Dose Modifications Based on Diarrhea in the OEP.

4.3.4. Retreatment Criteria

At the end of each cycle, subjects will have safety laboratory assessments performed to evaluate organ function. In order to proceed to the next cycle of treatment, subjects must continue to meet entry criteria regarding renal and hepatic function. At the end of each cycle, the start of the next cycle will be delayed if the subject does not meet the entry criteria for renal (serum creatinine ≤ 2.5 x ULN) or hepatic function (SGOT and SGPT ≤ 2.5 x ULN). Additionally, subjects who are evaluable for neutrophil and/or platelet toxicity must have an ANC > 500 µL, platelets > 25,000/µL, without evidence of a hypocellular marrow (> 5%). If abnormal renal, hepatic, and/or hematologic function persists for more than 21 days after detection, study treatment will be discontinued. Note that any nonhematologic toxic effect resulting in the delay of a subject's second cycle of oral azacitidine by > 14 days is considered a DLT. Subjects who are not evaluable for neutrophil and/or platelet toxicity (baseline ANC ≤ 500/µL and/or platelets ≤ 25,000/µL) will be treated as scheduled regardless of neutrophil and/or platelet count recovery. If retreatment criteria are not met following completion of the current cycle of treatment, the subject will be observed for up to an additional 4 weeks. If retreatment criteria are not met during these additional 4 weeks, the subject will be discontinued from protocol-prescribed therapy.

Retreatment criteria in OEP (Section **Error! Reference source not found.**) is similar to what is described above. In some circumstances, where delay in dosing may be due to other extraneous circumstances, the investigator should contact the medical monitor to discuss the reason to continue on therapy.

4.3.5. Intrasubject Dose Escalation

Intrasubject dose escalation of oral azacitidine will be permitted once a subject completes at least 2 cycles (subjects in Part 1) or 4 cycles (subjects in Part 2) of oral azacitidine therapy without experiencing significant drug-related toxicity, provided that the dose level to which the subject will escalate has already been evaluated and has a DLT rate ≤ 33% at the time of the proposed dose increase. The decision to escalate a subject's dose will be made between the Sponsor's Medical Monitor and Investigator prior to any discussion with the subject. There are no limits to the number of dose escalations a subject may have, provided the above considerations are met. However, only 1 dose escalation step per cycle will be permitted. Refer to Appendix M, Section **Error! Reference source not found.** for Intrasubject Dose Escalation in the OEP.

4.4. Method of Treatment Assignment

4.4.1. Part 1

All subjects will receive SC azacitidine followed by oral azacitidine treatment. The dose level for the oral treatment will be assigned by the Sponsor prior to dosing each subject with oral azacitidine.

4.4.2. Part 2

All subjects will receive treatment with oral azacitidine. The treatment schedule and dose level will be assigned by the Sponsor prior to dosing each subject with oral azacitidine.

4.4.3. OEP

Refer to Appendix M, Section **Error! Reference source not found.** for Method of Treatment Assignment in the OEP.

4.5. Blinding, Packaging, and Labeling

This is an open-label study. Study drug will be packaged by the Sponsor or its authorized representative and shipped to the clinic site in labeled containers. The container labels will include the drug name, dose strength, lot number, storage conditions, amount of azacitidine per container, and the manufacturer's name, as well as a statement that the drug is for clinical trial use only. Additional statements will be printed on the label(s) as required by local regulations.

4.6. Supplies and Accountability

The Investigator or pharmacist/designated personnel will inventory and acknowledge receipt of all shipments of study drug. All study drug must be kept in a locked area with access restricted to designated study personnel. The study drug must be stored at controlled temperature and a temperature log must be maintained. The Investigator or pharmacist/designated personnel will also keep accurate records of the quantities of study drug dispensed and used by each subject. For SC azacitidine, this includes the time of reconstitution, if refrigerated, and time of administration. The monitor will periodically check the supplies of study drug held by the Investigator or pharmacist/designated personnel to ensure accountability of all study drug used.

At the conclusion of the study, all remaining study drug containers will be counted, reconciled with dispensing records, documented, and returned to the Sponsor or designated representative. Alternatively, if directed by the Sponsor, at the completion of the study, unused drug may be destroyed at the clinic site after completion of drug accountability by the monitor. The monitor will assure that a final report of drug accountability to the unit dose level is prepared and placed in both the Investigator's Study File and the Trial Master File.

4.7. Compliance

Subcutaneous azacitidine will be administered by study site personnel. The date and time of administration will be documented in the subject's medical record and CRF.

Diary cards for use during oral azacitidine treatment will be provided by the Sponsor. Study site personnel will enter the scheduled daily dose of oral azacitidine in milligrams and the combination of tablets or capsules to be taken each day. Study site personnel will review the dosing information with the subject on scheduled clinic visit days. Subjects will be asked to record dosing information for oral azacitidine taken at home in the diary card and to bring the diary card and unused oral azacitidine with them to scheduled clinic visits. A compliance check and tablet or capsule count will be performed by study personnel. Study site personnel will record compliance information on the CRF and retain the diary card in the subject's medical record.

5. STUDY PROCEDURES

The procedures and assessments to be performed during Part 1 and Part 2 of the study are outlined in **Error! Reference source not found.**, **Error! Reference source not found.**, **Error! Reference source not found.**, and **Error! Reference source not found.**, and are described in the following sections. Actual times of dosing, bone marrow, blood and urine sample collection, and other assessments will be recorded in the subject's source documents and CRF.

Refer to Appendix M, Section **Error! Reference source not found.** for Study Procedures in the OEP.

5.1. Screening Procedures

Informed consent must be obtained prior to performing any study-specific tests or evaluations. Tests or evaluations performed as standard of care within the specified screening period, but prior to informed consent, may be accepted for this study upon approval by the subject and Sponsor's Medical Monitor. Proper documentation must be made in the subject's source documents.

The following screening assessments are required within 21 days prior to Cycle 1 Day 1, unless otherwise specified:

- Demographic data, including date of birth, gender, race, and smoking status (smoker or nonsmoker);
- Medical history, including major diseases and/or surgeries;
- Baseline signs and symptoms;
- Document all medications taken during the 4 weeks prior to screening and all transfusions administered during the 8 weeks prior to screening;
- Document type, number of units, reason, laboratory value prior to transfusion (eg, hemoglobin or platelet count) and date of each transfusion for low or Int-1 risk MDS given during the 8 weeks prior to screening;
- Physical examination, including height, body weight, and BSA calculated according to the formula of Mosteller. $BSA=([Weight (kg) x Height (cm)] / 3600)^{1/2}$;
- Diagnosis of low or Int-1 risk MDS according to IPSS (see Section **Error! Reference source not found.** and Section **Error! Reference source not found.**), which includes a bone marrow aspirate (or bone marrow biopsy, if aspirate cannot be obtained);
- ECOG performance status (see Section **Error! Reference source not found.**);
- International Prognostic Scoring System score (Section **Error! Reference source not found.**);
- 12-lead electrocardiogram (ECG), including rhythm, heart rate, and PR, QRS, and QT intervals;
- Vital signs, including systolic/diastolic blood pressure, pulse rate, respiratory rate, and oral temperature;

- Hematology
  - Complete blood count (CBC) including manual white blood cell (WBC) absolute differential and platelet count;
  - Red blood cell (RBC) indices (ie, mean corpuscular volume [MCV], mean corpuscular hemoglobin [MCH], and mean corpuscular hemoglobin concentration [MCHC]);
- Fasting serum chemistry
  - Electrolytes (sodium, potassium, calcium, phosphorous, bicarbonate, and chloride);
  - Renal function (BUN, creatinine, and uric acid);
  - Liver function (SGOT [also known as AST], SGPT [also known as ALT], total bilirubin, LDH, and alkaline phosphatase);
  - Other (glucose, albumin, and total protein);
- Serum vitamin B_{12} and folate;
- Coagulation evaluations, ie, International Normalized Ratio (INR), prothrombin time (PT), and partial thromboplastin time (PTT);
- Bone marrow aspirate for cytogenetic testing, clinical classification, and baseline PD obtained no more than 7 days prior to Cycle 1, Day 1 for Part 1 subjects and no more than 21 days prior to Cycle 1, Day 1 for Part 2 subjects;
- PD blood sampling obtained on the same day as the bone marrow procedure;
- Serum pregnancy test – if applicable for females of childbearing potential; obtained no more than 7 days prior to Cycle 1, Day 1; and
- Adverse event (AE) assessment (starting when the subject signs the informed consent).

5.2. On-Study Evaluations

Please refer to the Study Events Schedules. **Error! Reference source not found.** provides details for study procedures occurring from screening through Cycle 1 for subjects in Part 1. **Error! Reference source not found.** provides details for procedures occurring from Cycles 2 through 7, and for Cycles 8 and beyond for subjects in Part 1 who continue to receive oral azacitidine. **Error! Reference source not found.** provides details for procedures occurring during Cycles 1 through 6, and for Cycles 7 and beyond (if subject is continuing on oral azacitidine) for subjects participating in Part 2. **Error! Reference source not found.** provides details for procedures occurring during Cycles 8 and beyond (subjects in Part 1) and during Cycles 7 and beyond (subjects in Part 2) in subjects crossed over to SC azacitidine. A ± 2-day window is permitted for all study visits, unless otherwise specified.

Subjects in Part 1 should be advised not to consume grapefruit/grapefruit juice for 3 days prior to Day 1 and for 15 days after Day 1. Subjects in Part 1 may have grapefruit/grapefruit juice during Day 16 through Day 25. Subjects in Part 2 should be advised not to consume

grapefruit/grapefruit juice during for 3 days prior to Cycle 1, Day 1 and should refrain from any grapefruit/grapefruit juice consumption while receiving oral azacitidine. Any subject who crosses over to SC azacitidine should not consume grapefruit/grapefruit juice for 3 days prior to Day 1 of the cycle and for 15 days after Day 1 of each cycle. All subjects should be instructed to limit their coffee intake to one 8 ounce cup of black coffee (no cream or sugar) and to not consume alcohol, tea, chocolate, or cola beverages within 2 hours prior to collection of PK samples.

5.2.1. On-Study Evaluations for Subjects in Part 1

Cycle 1, Day 1

All subjects will report to the clinic for the following procedures, unless the procedures have been done within the last 3 days:

- Document concomitant medications and transfusions;
- AE assessment;
- Baseline signs and symptoms;
- Physical examination, including height, body weight and BSA calculated according to the formula of Mosteller. BSA=([Weight (kg) x Height (cm)] / 3600)$^{1/2}$;
- Vital signs, including blood pressure, pulse rate, respiratory rate, and oral temperature;
- 12-lead ECG;
- ECOG Performance Score;
- Hematology and serum chemistry as defined in Section 6.2.3.2;
- Blood sample for PK analysis, at predose, and at 0.25, 0.5, 1, 1.5, 2, 2.5, 3, 3.5, 4, 6, and 8 hours after dosing;
- Urine samples for PK analysis at predose (single void up to 1 hour before dosing) and for all voids thereafter up to 8 hours after dosing. Record the time and volume of each void. Freeze 2 aliquots (as per PK/PD Laboratory Manual) and discard the remaining urine;
- Approximately 30 minutes prior to dose, administer an antiemetic; and
- Administer SC azacitidine.

Cycle 1, Day 2

All subjects will report to the clinic for the following procedures:

- Document concomitant medications and transfusions;
- AE assessment;
- Approximately 30 minutes prior to dose, administer an antiemetic; and
- Administer SC azacitidine.

Cycle 1, Day 3

All subjects will report to the clinic for the following procedures:

- Document concomitant medications and transfusions;
- AE assessment;
- PD blood sampling at predose;
- Approximately 30 minutes prior to dose, administer an antiemetic; and
- Administer SC azacitidine.

Cycle 1, Days 4, 5, and 6

All subjects will return to the clinic for the following procedures:

- Document concomitant medications and transfusions;
- AE assessment;
- Approximately 30 minutes prior to dose, administer an antiemetic;
- Administer SC azacitidine; and
- Day 6, remind subjects to limit their coffee intake to one 8 ounce cup of black coffee (no cream or sugar) and to not consume alcohol, tea, chocolate or cola beverages within 2 hours prior to dosing on Cycle 1, Day 7. Water and any required medications are permitted during the fasting period.

Cycle 1, Day 7

All subjects will report to the clinic for the following procedures:

- Document concomitant medications and transfusions;
- AE assessment;
- Blood sample for PK analysis at predose and at 0.25, 0.5, 1, 1.5, 2, 2.5, 3, 3.5, 4, 6, and 8 hours after dosing;
- Urine samples for PK analysis at predose (single void up to 1 hour before dosing) and for all voids thereafter up to 8 hours after dosing. Record the time and volume of each void. Freeze 2 aliquots (as per Laboratory Manual) and discard the remaining urine;
- Approximately 30 minutes prior to dose, administer an antiemetic; and
- Administer SC azacitidine.

Cycle 1, Day 8

All subjects will report to the clinic for the following procedures:

- Document concomitant medications and transfusions;
- AE assessment;
- Hematology as defined in Section 6.2.3.2;

- PD blood sampling; and
- Bone marrow aspirate (or bone marrow biopsy, if aspirate cannot be obtained) collected ± 3 days.

Cycle 1, Days 15 and 22

All subjects will report to the clinic for the following procedures:

- Document concomitant medications and transfusions;
- AE assessment;
- Hematology as defined in Section 6.2.3.2; and
- PD blood sampling.

Cycle 1, Day 28

All subjects will report to the clinic for the following procedures:

- Document concomitant medications and transfusions;
- AE assessment;
- Hematology and serum chemistry as defined in Section 6.2.3.2;
- Revised IWG hematologic response and/or improvement assessment (see Appendix D, Section **Error! Reference source not found.**; Appendix I, Section **Error! Reference source not found.**; and Appendix J, Section **Error! Reference source not found.**); and
- Subjects should be instructed to limit their coffee intake to one 8 ounce cup of black coffee (no cream or sugar) on Day 1 and not consume alcohol, tea, chocolate, or cola beverages within 2 hours prior to Cycle 2, Day 1. Subjects will need to fast for a minimum of 2 hours prior to ingesting oral azacitidine at the Cycle 2, Day 1 clinic visit. Water and any required medications are permitted during the fasting period.

Note: A bone marrow aspirate, CBC and PD blood samples are required no more than 7 days prior to Cycle 2, Day 1. Appropriate laboratory assessments must be performed on, or within 3 days prior to Cycle 2, Day 1 in order to ensure toxicities experienced in Cycle 1 have resolved and the subject meets the retreatment criteria as described in Section 7.

Cycle 2, Day 1

All subjects will report to the clinic for the following procedures:

- Document concomitant medications and transfusions;
- AE assessment;
- Physical examination;
- Body weight;
- Vital signs, including blood pressure, pulse rate, respiratory rate, and oral temperature;

- 12-lead ECG;
- ECOG Performance Score;
- Hematology and serum chemistry as defined in Section 6.2.3.2 unless Day 1 is within 3 days of Cycle 1, Day 28. If so, then a sample does not need to be collected;
- Urine or serum pregnancy test – if applicable for females of childbearing potential; obtained no more than 7 days prior to Cycle 2, Day 1;
- Blood sample for PK analysis, at predose, and at 0.25, 0.5, 1, 1.5, 2, 2.5, 3, 3.5, 4, 6, and 8 hours after dosing;
- Bone marrow aspirate, CBC and PD blood sampling collected no more than 7 days prior to Cycle 2 Day 1;
- Urine samples for PK analysis at predose (single void up to 1 hour before dosing) and for all voids thereafter up to 8 hours after dosing. Record the time and volume of each void. Freeze 2 aliquots (as per Laboratory Manual) and discard the remaining urine;
- Approximately 30 minutes prior to dose, administer an antiemetic;
- After a 2-hour fast, administer oral azacitidine with the minimum of 8 ounces (240 mL) of room temperature water in the clinic. Please record the exact time of dosing and the amount of water ingested. Remind subjects to fast for 2 hours after taking oral azacitidine; and
- Dispense azacitidine tablets or capsules and antiemetic for Cycle 2, Day 2 dosing, and instruct subjects on the use of the diary card.

Subjects will self-administer antiemetic and oral azacitidine at home on Day 2.

Cycle 2, Day 3

All subjects will report to the clinic for the following procedures:

- Diary card review;
- Document concomitant medications and transfusions;
- AE assessment;
- PD blood sampling at predose;
- Approximately 30 minutes prior to dose, administer antiemetic;
- After a 2-hour fast, administer oral azacitidine with the minimum of 8 ounces (240 mL) of room temperature water in the clinic. Please record the exact time of dosing and the amount of water ingested. Remind subjects to fast for 2 hours after taking oral azacitidine;
- Dispense azacitidine tablets or capsules and antiemetic for Cycle 2, Days 4, 5, and 6 dosing, and redispense diary card. Study drug will be self-administered at home on treatment days not scheduled at the clinic; and

- Subjects should be instructed to limit their coffee intake to one 8 ounce cup of black coffee (no cream or sugar) on Day 7 and not consume alcohol, tea, chocolate, or cola beverages within 2 hours prior to Cycle 2, Day 7. Subjects will need to fast for a minimum of 2 hours prior to ingesting oral azacitidine at the Cycle 2, Day 7 clinic visit. Water and any required medications are permitted during the fasting period.

Subjects will self-administer antiemetic and oral azacitidine at home on Days 4, 5, and 6.

Cycle 2, Day 7

All subjects will report to the clinic for the following procedures:

- Collect unused oral azacitidine;
- Diary card review;
- Document concomitant medications and transfusions;
- AE assessment;
- Blood sample for PK analysis at predose and at 0.25, 0.5, 1, 1.5, 2, 2.5, 3, 3.5, 4, 6, and 8 hours after dosing;
- Urine samples for PK analysis at predose (single void up to 1 hour before dosing) and for all voids thereafter up to 8 hours after dosing. Record the time and volume of each void. Freeze 2 aliquots (as per Laboratory Manual) and discard the remaining urine;
- Approximately 30 minutes prior to dose, administer antiemetic; and
- After a 2-hour fast, administer oral azacitidine with the minimum of 8 ounces (240 mL) of room temperature water in the clinic. Please record the exact time of dosing and the amount of water ingested. Remind subjects to fast for 2 hours after taking oral azacitidine.

Cycle 2, Day 8

All subjects will report to the clinic for the following procedures:

- Document concomitant medications and transfusions;
- AE assessment;
- Hematology as defined in Section 6.2.3.2;
- PD blood sampling; and
- Bone marrow aspirate (or bone marrow biopsy, if aspirate cannot be obtained) collected ± 3 days.

Cycle 2, Days 15 and 22

All subjects will report to the clinic for the following procedures:

- Document concomitant medications and transfusions;
- AE assessment;

- Hematology as defined in Section 6.2.3.2; and
- PD blood sampling.

Cycle 2, Day 28

All subjects will report to the clinic for the following procedures:

- Document concomitant medications and transfusions;
- AE assessment;
- Hematology and serum chemistry as defined in Section 6.2.3.2;
- Revised IWG hematologic response and/or improvement assessment (see Appendix D, Section **Error! Reference source not found.**; Appendix I, Section **Error! Reference source not found.**; and Appendix J, Section **Error! Reference source not found.**); and

Note: If the start of Cycle 3 is delayed by > 7 days following the Cycle 2, Day 28 assessment, appropriate laboratory assessments must be performed on, or immediately prior to, Cycle 3, Day 1 in order to ensure toxicities experienced in Cycle 2 have resolved and the subject meets the retreatment criteria as described in Section 7.

Cycle 3, Day 1

All subjects will report to the clinic for the following procedures:

- Document concomitant medications and transfusions;
- AE assessment;
- Physical examination;
- Body weight;
- Vital signs, including blood pressure, pulse rate, respiratory rate, and oral temperature;
- 12-lead ECG;
- ECOG Performance Score;
- Hematology as defined in Section 6.2.3.2 unless Day 1 is within 3 days of Cycle 2, Day 28. If so, then a sample does not need to be collected;
- Serum or urine pregnancy test - if applicable for females of childbearing potential; obtained no more than 7 days prior to Day 1 of the next cycle;
- PD blood sampling at predose;
- Approximately 30 minutes prior to dose, administer an antiemetic;
- Administer oral azacitidine with the minimum of 8 ounces (240 mL) of room temperature water in the clinic. Please record the exact time of dosing and the amount of water ingested. Oral azacitidine may be taken on an empty stomach or with food. If the dose is taken in the morning, subjects may consume their usual breakfast before or after administration. The meal should not exceed 600 calories;

however, the actual calorie count will not need to be measured or recorded. If a meal other than breakfast is consumed, a light meal (not more than 25% of a subject's usual daily calories) may be eaten before or after dose administration; and

- Dispense azacitidine tablets or capsules, antiemetic, and diary card to subjects receiving oral azacitidine. Study drug will be self-administered at home on treatment days not scheduled at the clinic.

Subjects will self-administer antiemetic and oral azacitidine at home on Days 2, 3, 4, 5, 6, and 7 of Cycle 3.

Cycle 3, Days 15 and 28

All subjects will report to the clinic for the following procedures:

- Collect unused oral azacitidine (Day 15 only);
- Diary card review (Day 15 only);
- Document concomitant medications and transfusions;
- AE assessment;
- PD blood sampling (Day 15 only);
- Hematology as defined in Section 6.2.3.2 (Days 15 and 28);
- Serum chemistry (Day 28 only); and
- Revised IWG hematologic response and/or improvement assessment (see Appendix D, Section **Error! Reference source not found.**; Appendix I, Section **Error! Reference source not found.**; and Appendix J, Section **Error! Reference source not found.**) (Day 28 only).

Cycles 4, 5 and 6, Day 1

All subjects will report to the clinic for the following procedures:

- Document concomitant medications and transfusions;
- AE assessment;
- Physical examination;
- Body weight;
- Vital signs, including blood pressure, pulse rate, respiratory rate, and oral temperature;
- 12-lead ECG;
- ECOG Performance Score;
- Hematology as defined in Section 6.2.3.2 unless Day 1 is within 3 days of Day 28 of the previous cycle. If so, then a sample does not need to be collected;
- Serum or urine pregnancy test - if applicable for females of childbearing potential; obtained no more than 7 days prior to Day 1 of the next cycle;

- PD blood sampling at predose;
- Approximately 30 minutes prior to dose, administer an antiemetic;
- Administer oral azacitidine with the minimum of 8 ounces (240 mL) of room temperature water in the clinic. Please record the exact time of dosing and the amount of water ingested. Oral azacitidine may be taken on an empty stomach or with food. If the dose is taken in the morning, subjects may consume their usual breakfast before or after administration. The meal should not exceed 600 calories; however, the actual calorie count will not need to be measured or recorded. If a meal other than breakfast is consumed, a light meal (not more than 25% of a subject's usual daily calories) may be eaten before or after dose administration; and
- Dispense azacitidine tablets or capsules, antiemetic, and diary card. Study drug will be self-administered at home on treatment days not scheduled at the clinic.

Subjects will self-administer antiemetic and oral azacitidine at home on Days 2, 3, 4, 5, 6, and 7.

Cycles 4, 5 and 6, Days 15 and 28

All subjects will report to the clinic for the following procedures:

- Collect unused oral azacitidine (Day 15 only);
- Diary card review (Day 15 only);
- PD blood sampling (Day 15 only);
- Document concomitant medications and transfusions;
- AE assessment;
- Hematology as defined in Section 6.2.3.2 (Days 15 and 28);
- Serum chemistry (Day 28 only); and
- Revised IWG hematologic response and/or improvement assessment (see Appendix D, Section **Error! Reference source not found.**; Appendix I, Section **Error! Reference source not found.**; and Appendix J, Section **Error! Reference source not found.**) (Day 28 only).

Cycle 7, Day 1

All subjects will report to the clinic for the following procedures:

- Document concomitant medications and transfusions;
- AE assessment;
- Physical examination;
- Body weight;
- Vital signs, including blood pressure, pulse rate, respiratory rate, and oral temperature;
- 12-lead ECG;

- ECOG Performance Score;
- Hematology as defined in Section 6.2.3.2 unless Day 1 is within 3 days of Cycle 6, Day 28. If so, then a sample does not need to be collected;
- Serum or urine pregnancy test - if applicable for females of childbearing potential; obtained no more than 7 days prior to Day 1 of the next cycle;
- PD blood sampling at predose;
- Approximately 30 minutes prior to dose, administer an antiemetic;
- Administer oral azacitidine with the minimum of 8 ounces (240 mL) of room temperature water in the clinic. Please record the exact time of dosing and the amount of water ingested. Oral azacitidine may be taken on an empty stomach or with food. If the dose is taken in the morning, subjects may consume their usual breakfast before or after administration. The meal should not exceed 600 calories; however, the actual calorie count will not need to be measured or recorded. If a meal other than breakfast is consumed, a light meal (not more than 25% of a subject's usual daily calories) may be eaten before or after dose administration; and
- Dispense azacitidine tablets or capsules, antiemetic, and diary card. Study drug will be self-administered at home on treatment days not scheduled at the clinic.

Subjects will self-administer antiemetic and oral azacitidine at home on Days 2, 3, 4, 5, and 6.

Cycle 7, Day 7

All subjects will report to the clinic for the following procedures:

- Collect unused oral azacitidine;
- Diary card review;
- AE assessment;
- PD blood sampling at pre-dose;
- Blood sample for PK analysis at predose and at 0.25, 0.5, 1, 1.5, 2, 2.5, 3, 3.5, 4, 6, and 8 hours after dosing;
- Approximately 30 minutes prior to dose, administer antiemetic; and
- After a 2-hour fast, administer oral azacitidine with the minimum of 8 ounces (240 mL) of room temperature water in the clinic. Please record the exact time of dosing and the amount of water ingested. Remind subjects to fast for 2 hours after taking oral azacitidine.

Cycle 7, Days 15 and 28

All subjects will report to the clinic for the following procedures:

- Document concomitant medications and transfusions;
- AE assessment;
- Hematology as defined in Section 6.2.3.2 (Days 15 and 28);

- Serum chemistry (Day 28 only);
- Bone marrow aspirate and PD blood sampling collected on Cycle 7, Day 28 (± 7 day window);
- Cytogenetic testing on bone marrow aspirate if collected (Day 28); and
- Revised IWG hematologic response and/or improvement assessment (see Appendix D, Section **Error! Reference source not found.**; Appendix I, Section **Error! Reference source not found.**; and Appendix J, Section **Error! Reference source not found.**) (Day 28 only).

Note: Additional bone marrow aspirates should be obtained as clinically indicated. PD blood and bone marrow sampling should accompany clinically indicated/unscheduled procedures.

At the end of Cycle 7, the subject will have completed protocol-specified treatment. Subjects who respond may continue receiving treatment with oral azacitidine. If the subject is not responding, the subject may stay on oral azacitidine (possible dose increase) or may cross over to SC azacitidine. It is the Investigator's decision to continue treating the subject with either oral or SC azacitidine. The procedures and assessments for Cycles 8 and beyond for subjects on oral azacitidine are outlined immediately below.

Cycles 8 and beyond, Day 1 for subjects on oral azacitidine

All subjects will report to the clinic for the following procedures:

- Document concomitant medications and transfusions;
- AE assessment;
- Physical examination;
- Body weight;
- Vital signs, including blood pressure, pulse rate, respiratory rate, and oral temperature;
- 12-lead ECG;
- ECOG Performance Score;
- Hematology as defined in Section 6.2.3.2 unless Day 1 is within 3 days of Day 28 of the previous cycle. If so, then a sample does not need to be collected;
- Serum or urine pregnancy test - if applicable for females of childbearing potential; obtained no more than 7 days prior to Day 1 of the next cycle;
- PD blood sampling at predose;
- Approximately 30 minutes prior to dose, administer an antiemetic;
- Administer oral azacitidine with the minimum of 8 ounces (240 mL) of room temperature water in the clinic. Please record the exact time of dosing and the amount of water ingested. Oral azacitidine may be taken on an empty stomach or with food. If the dose is taken in the morning, subjects may consume their usual breakfast before or after administration. The meal should not exceed 600 calories;

however, the actual calorie count will not need to be measured or recorded. If a meal other than breakfast is consumed, a light meal (not more than 25% of a subject's usual daily calories) may be eaten before or after dose administration; and

- Dispense azacitidine tablets or capsules, antiemetic, and diary card. Study drug will be self-administered at home on treatment days not scheduled at the clinic.

Subjects will self-administer antiemetic and oral azacitidine at home on Days 2, 3, 4, 5, 6, and 7.

Cycles 8 and beyond, Days 15 and 28 for subjects on oral azacitidine

All subjects will report to the clinic for the following procedures:

- Collect unused oral azacitidine (Day 15 only);
- Diary card review (Day 15 only);
- Document concomitant medications and transfusions;
- AE assessment;
- Hematology as defined in Section 6.2.3.2 (Days 15 and 28);
- Serum chemistry (Day 28 only); and
- Revised IWG hematologic response and/or improvement assessment (see Appendix D, Section **Error! Reference source not found.**; Appendix I, Section **Error! Reference source not found.**; and Appendix J, Section **Error! Reference source not found.**) (Day 28 only).

Cycles 8 and beyond, Day 1 for subjects crossed over to SC azacitidine

Subjects who cross over to SC azacitidine should not consume grapefruit/grapefruit juice for 3 days prior to Day 1 of the cycle and for 15 days after Day 1 of each cycle.

All subjects will report to the clinic for the following procedures:

- Document concomitant medications and transfusions;
- AE assessment;
- Physical examination, including height, body weight and BSA calculated according to the formula of Mosteller. BSA=([Weight (kg) x Height (cm)] / 3600)$^{1/2}$;
- Vital signs, including blood pressure, pulse rate, respiratory rate, and oral temperature;
- 12-lead ECG;
- ECOG Performance Score;
- Hematology as defined in Section 6.2.3.2 unless Day 1 is within 3 days of Day 28 of the previous cycle. If so, then a sample does not need to be collected;
- Serum chemistry (Cycle 8 only) as defined in Section 6.2.3.2;
- Serum or urine pregnancy test - if applicable for females of childbearing potential; obtained no more than 7 days prior to Day 1 of the next cycle;

- PD blood sampling at predose;
- Approximately 30 minutes prior to dose, administer an antiemetic; and
- Administer SC azacitidine in the clinic**.**

Cycles 8 and beyond, Days 2, 3, 4, 5, 6 and 7 for subjects crossed over to SC azacitidine

All subjects will report to the clinic for the following procedures:

- Document concomitant medications and transfusions;
- AE assessment;
- Approximately 30 minutes prior to dose, administer an antiemetic; and
- Administer SC azacitidine.

Cycles 8 and beyond, Day 15 for subjects crossed over to SC azacitidine

All subjects will report to the clinic for the following procedures:

- Document concomitant medications and transfusions;
- AE assessment; and
- Hematology as defined in Section 6.2.3.2.

Cycles 8 and beyond, Day 28 for subjects crossed over to SC azacitidine

All subjects will report to the clinic for the following procedures:

- Document concomitant medications and transfusions;
- AE assessment;
- Hematology and serum chemistry as defined in Section 6.2.3.2; and
- Revised IWG hematologic response and/or improvement assessment (see Appendix D, Section **Error! Reference source not found.**; Appendix I, Section **Error! Reference source not found.**; and Appendix J, Section **Error! Reference source not found.**).

5.2.2. On-Study Evaluations for Subjects in Part 2

Cycles 1 and 2, Day 1

On Cycle 1, Day 1, all subjects will report to the clinic for the following procedures, unless the procedures have been done within the last 3 days. On Cycle 2, Day 1, all subjects will report to the clinic for the following procedures:

- Baseline signs and symptoms (Cycle 1 only);
- Physical examination, body weight, and BSA calculated according to the formula of Mosteller. BSA=([Weight (kg) x Height (cm)] / 3600)^{½} (BSA at Cycle 1 only);
- Vital signs, including blood pressure, pulse rate, respiratory rate, and oral temperature;

- ECOG Performance Score;
- 12-lead ECG;
- Document concomitant medications and transfusions;
- Hematology and serum chemistry as defined in Section 6.2.3.2;
- Serum or urine pregnancy test - if applicable for females of childbearing potential; obtained no more than 7 days prior to Day 1 of the next cycle;
- PD blood sampling at predose;
- Approximately 30 minutes prior to dose, administer an antiemetic;
- Administer oral azacitidine with the minimum of 8 ounces (240 mL) of room temperature water in the clinic. Please record the exact time of dosing and the amount of water ingested. Oral azacitidine may be taken on an empty stomach or with food. If the dose is taken in the morning, subjects may consume their usual breakfast before or after administration. The meal should not exceed 600 calories; however, the actual calorie count will not need to be measured or recorded. If a meal other than breakfast is consumed, a light meal (not more than 25% of a subject's usual daily calories) may be eaten before or after dose administration; and
- Dispense azacitidine tablets or capsules and antiemetic for Days 2-14 or Days 2-21 of cycle, depending on the subject's treatment schedule, and instruct subjects on the use of the diary card; and
- AE assessment.

Subjects will self-administer antiemetic and oral azacitidine at home on Days 2-7.

Cycles 1 and 2, Day 8

All subjects will report to the clinic for the following procedures:

- Document concomitant medications and transfusions;
- Hematology as defined in Section 6.2.3.2;
- PD blood sampling; and
- AE assessment.

Subjects may self-administer antiemetic and oral azacitidine at home or in the clinic on Day 8.

Subjects will self-administer antiemetic and oral azacitidine at home on Days 9 – 13.

Oral azacitidine may be taken on an empty stomach or with food. If the dose is taken in the morning, subjects may consume their usual breakfast before or after administration. The meal should not exceed 600 calories; however, the actual calorie count will not need to be measured or recorded. If a meal other than breakfast is consumed, a light meal (not more than 25% of a subject's usual daily calories) may be eaten before or after dose administration.

Cycles 1 and 2, Day 14

All subjects will report to the clinic for the following procedures:

- Document concomitant medications and transfusions;
- Hematology and serum chemistry as defined in Section 6.2.3.2;
- PD blood sampling;
- Collect unused oral azacitidine (subjects on a14-day QD treatment schedule);
- Diary card review (subjects on a 14-day QD treatment schedule); and
- AE assessment.

Subjects on a 14-day treatment schedule will take their antiemetic and oral azacitidine at home or in the clinic.

Subjects on a 21-day treatment schedule may self-administer antiemetic and oral azacitidine in the clinic or at home on Day 14, and will self-administer antiemetic and oral azacitidine at home on Days 15 – 20.

Oral azacitidine may be taken on an empty stomach or with food. If the dose is taken in the morning, subjects may consume their usual breakfast before or after administration. The meal should not exceed 600 calories; however, the actual calorie count will not need to be measured or recorded. If a meal other than breakfast is consumed, a light meal (not more than 25% of a subject's usual daily calories) may be eaten before or after dose administration.

Cycles 1 and 2, Day 21

All subjects will report to the clinic for the following procedures:

- Document concomitant medications and transfusions;
- Hematology as defined in Section 6.2.3.2;
- Bone marrow aspirate (or bone marrow biopsy, if aspirate cannot be obtained) collected ± 2 days;
- PD blood sampling;
- Collect unused oral azacitidine (subjects on a 14-day BID or 21-day QD treatment schedule);
- Diary card review (subjects on a 14-day BID or 21-day QD treatment schedule); and
- AE assessment.

Subjects on a 21-day treatment schedule will take their antiemetic and oral azacitidine at home or in the clinic.

Oral azacitidine may be taken on an empty stomach or with food. If the dose is taken in the morning, subjects may consume their usual breakfast before or after administration. The meal should not exceed 600 calories; however, the actual calorie count will not need to be measured or recorded. If a meal other than breakfast is consumed, a light meal (not more than 25% of a subject's usual daily calories) may be eaten before or after dose administration.

Cycles 1 and 2, Day 28

All subjects will report to the clinic for the following procedures:

- Document concomitant medications and transfusions;
- Hematology and serum chemistry as defined in Section 6.2.3.2;
- Collect unused oral azacitidine (subjects on a 21-day BID treatment schedule);
- Diary card review (subjects in the 21-day BID treatment schedule);
- Revised IWG hematologic response and/or improvement assessment (see Appendix D, Section **Error! Reference source not found.**; Appendix I, Section **Error! Reference source not found.**; and Appendix J, Section **Error! Reference source not found.**) (Cycle 2 only); and
- AE assessment.

Cycles 3, 4, 5, and 6, Day 1

All subjects will report to the clinic for the following procedures:

- Physical examination;
- Vital signs, including blood pressure, pulse rate, respiratory rate, and oral temperature;
- Body weight;
- ECOG Performance Score;
- 12-lead ECG;
- Document concomitant medications and transfusions;
- Hematology as defined in Section 6.2.3.2 unless Day 1 is within 3 days of Day 28 of the previous cycle. If so, then a sample does not need to be collected;
- Serum or urine pregnancy test - if applicable for females of childbearing potential; obtained no more than 7 days prior to Day 1 of the next cycle;
- PD blood sampling at predose;
- Approximately 30 minutes prior to dose, administer an antiemetic;
- Administer oral azacitidine with the minimum of 8 ounces (240 mL) of room temperature water in the clinic. Please record the exact time of dosing and the amount of water ingested. Oral azacitidine may be taken on an empty stomach or with food. If the dose is taken in the morning, subjects may consume their usual breakfast before or after administration. The meal should not exceed 600 calories; however, the actual calorie count will not need to be measured or recorded. If a meal other than breakfast is consumed, a light meal (not more than 25% of a subject's usual daily calories) may be eaten before or after dose administration; and
- Dispense azacitidine tablets or capsules, antiemetic, and diary card to subjects receiving oral azacitidine. Study drug will be self-administered at home on treatment days not scheduled at the clinic; and
- AE assessment.

Note: If the start of a cycle is delayed by > 7 days following the Day 28 assessment of the previous cycle, appropriate laboratory assessments must be performed on, or immediately prior to, Day 1 in order to ensure toxicities experienced in the previous cycle have resolved and the subject meets the retreatment criteria as described in Section 7.

Subjects will self-administer antiemetic and oral azacitidine at home on Days 2 – 13.

Cycles 3, 4, 5, and 6, Day 14

All subjects will report to the clinic for the following procedures:

- Document concomitant medications and transfusions;
- Hematology and serum chemistry as defined in Section 6.2.3.2;
- Collect unused oral azacitidine (subjects on a 14-day QD treatment schedule);
- Diary card review (subjects on a 14-day QD treatment schedule); and
- AE assessment.

Subjects will self-administer antiemetic and oral azacitidine at home or in the clinic on Day 14.

Subjects on a 21-day treatment schedule will self-administer antiemetic and oral azacitidine at home on Days 15 – 20.

Oral azacitidine may be taken on an empty stomach or with food. If the dose is taken in the morning, subjects may consume their usual breakfast before or after administration. The meal should not exceed 600 calories; however, the actual calorie count will not need to be measured or recorded. If a meal other than breakfast is consumed, a light meal (not more than 25% of a subject's usual daily calories) may be eaten before or after dose administration.

Cycles 3, 4, 5, and 6, Day 21

Subjects on a 21-day treatment schedule will self-administer antiemetic and oral azacitidine at home or in the clinic on Day 21.

Oral azacitidine may be taken on an empty stomach or with food. If the dose is taken in the morning, subjects may consume their usual breakfast before or after administration. The meal should not exceed 600 calories; however, the actual calorie count will not need to be measured or recorded. If a meal other than breakfast is consumed, a light meal (not more than 25% of a subject's usual daily calories) may be eaten before or after dose administration.

Cycles 3, 4, 5, and 6, Day 28

All subjects will report to the clinic for the following procedures:

- Document concomitant medications and transfusions;
- Hematology and serum chemistry as defined in Section 6.2.3.2;
- Bone marrow aspirate (or bone marrow biopsy, if aspirate cannot be obtained) collected on:
  - Cycle 4, Day 28 (± 3 days)
  - Cycle 6, Day 28 (± 3 days)

- Cytogenetic testing on bone marrow aspirate if collected (Cycle 6, Day 28 only);
- Collect unused oral azacitidine (subjects on a 14-day BID, 21-day QD, or 21-day BID treatment schedule);
- Diary card review (subjects on a 14-day BID, 21-day QD, or 21-day BID treatment schedule);
- AE assessment; and
- Revised IWG hematologic response and/or improvement assessment (see Appendix D, Section **Error! Reference source not found.**; Appendix I, Section **Error! Reference source not found.**; and Appendix J, Section **Error! Reference source not found.**) (Cycle 4 and Cycle 6 only)

Note: Additional bone marrow aspirates should be obtained as clinically indicated. PD blood and bone marrow sampling should accompany clinically indicated/unscheduled procedures.

At the end of Cycle 6, the subject will have completed protocol-specified treatment. Subjects who respond may continue receiving treatment with oral azacitidine. If the subject is not responding, the subject may stay on oral azacitidine (possible dose increase) or may cross over to SC azacitidine. It is the Investigator's decision to continue treating the subject with either oral or SC azacitidine.

Cycles 7 and beyond, Day 1 for subjects on oral azacitidine

All subjects will report to the clinic for the following procedures:

- Physical examination;
- Vital signs, including blood pressure, pulse rate, respiratory rate, and oral temperature;
- Body weight;
- ECOG Performance Score;
- 12-lead ECG;
- Document concomitant medications and transfusions;
- Hematology as defined in Section 6.2.3.2 unless Day 1 is within 3 days of Day 28 of the previous cycle. If so, then a sample does not need to be collected;
- Serum or urine pregnancy test - if applicable for females of childbearing potential; obtained no more than 7 days prior to Day 1 of the next cycle;
- PD blood sampling at predose;
- AE assessment;
- Approximately 30 minutes prior to dose, administer an antiemetic;
- Administer oral azacitidine with the minimum of 8 ounces (240 mL) of room temperature water in the clinic. Please record the exact time of dosing and the amount of water ingested. Oral azacitidine may be taken on an empty stomach or

with food. If the dose is taken in the morning, subjects may consume their usual breakfast before or after administration. The meal should not exceed 600 calories; however, the actual calorie count will not need to be measured or recorded. If a meal other than breakfast is consumed, a light meal (not more than 25% of a subject's usual daily calories) may be eaten before or after dose administration; and

- Dispense azacitidine tablets or capsules, antiemetic, and diary card. Study drug will be self-administered at home on treatment days not scheduled at the clinic.

Subjects will self-administer antiemetic and oral azacitidine at home on Days 2 – 14 or Days 2 – 21, depending on a subject's treatment schedule.

Oral azacitidine may be taken on an empty stomach or with food. If the dose is taken in the morning, subjects may consume their usual breakfast before or after administration. The meal should not exceed 600 calories; however, the actual calorie count will not need to be measured or recorded. If a meal other than breakfast is consumed, a light meal (not more than 25% of a subject's usual daily calories) may be eaten before or after dose administration.

Cycles 7 and beyond, Days 14 and 28 for subjects on oral azacitidine

All subjects will report to the clinic for the following procedures:

- Collect unused oral azacitidine (Day 14 for subjects on a 14-day QD schedule and Day 28 for subjects on a 14-day BID, 21-day QD, or 21-day BID treatment schedule);
- Diary card review (Day 14 for subjects on a 14-day QD schedule and Day 28 for subjects on a 14-day BID, 21-day QD, or 21-day BID treatment schedule);
- Document concomitant medications and transfusions;
- AE assessment;
- Hematology as defined in Section 6.2.3.2;
- Serum chemistry (Day 28 only); and
- Revised IWG hematologic response and/or improvement assessment (see Appendix D, Section **Error! Reference source not found.**; Appendix I, Section **Error! Reference source not found.**; and Appendix J, Section **Error! Reference source not found.**).(Day 28 of every 2nd cycle only, beginning at Cycle 8).

Cycles 7 and beyond, Day 1 for subjects crossed over to SC azacitidine

Subjects who cross over to SC azacitidine should not consume grapefruit/grapefruit juice for 3 days prior to Day 1 of the cycle and for 15 days after Day 1 of each cycle.

All subjects will report to the clinic for the following procedures:

- Document concomitant medications and transfusions;
- AE assessment;
- Physical examination, including height, body weight and BSA calculated according to the formula of Mosteller. BSA=([Weight (kg) x Height (cm)] / 3600)$^{1/2}$;

- Vital signs, including blood pressure, pulse rate, respiratory rate, and oral temperature;
- 12-lead ECG;
- ECOG Performance Score;
- Hematology as defined in Section 6.2.3.2 unless Day 1 is within 3 days of Day 28 of the previous cycle. If so, then a sample does not need to be collected;
- Serum chemistry (Cycle 8 only) as defined in Section 6.2.3.2;
- Serum or urine pregnancy test - if applicable for females of childbearing potential; obtained no more than 7 days prior to Day 1 of the next cycle;
- PD blood sampling at predose;
- Approximately 30 minutes prior to dose, administer an antiemetic; and
- Administer SC azacitidine in the clinic.

Cycles 7 and beyond, Days 2, 3, 4, 5, 6 and 7 for subjects crossed over to SC azacitidine

All subjects will report to the clinic for the following procedures:

- Document concomitant medications and transfusions;
- AE assessment;
- Approximately 30 minutes prior to dose, administer an antiemetic; and
- Administer SC azacitidine.

Cycles 7 and beyond, Day 15 for subjects crossed over to SC azacitidine

All subjects will report to the clinic for the following procedures:

- Document concomitant medications and transfusions;
- AE assessment; and
- Hematology as defined in Section 6.2.3.2.

Cycles 7 and beyond, Day 28 for subjects crossed over to SC azacitidine

All subjects will report to the clinic for the following procedures:

- Document concomitant medications and transfusions;
- AE assessment;
- Hematology and serum chemistry as defined in Section 6.2.3.2; and
- Revised IWG hematologic response and/or improvement assessment (see Appendix D, Section **Error! Reference source not found.**; Appendix I, Section **Error! Reference source not found.**; and Appendix J, Section **Error! Reference source not found.**).

5.2.3. Optional Extension Phase

Refer to Appendix M, Section **Error! Reference source not found.** for Study Procedures in the OEP.

5.3. End of Study/Subject Discontinuation

The end of study visit from Part 1 or Part 2 will occur 28 days after the last dose of study drug, at study withdrawal, even if the date does not correspond to a protocol-specified visit, or within 21 days of entering the OEP. For subjects lost to follow-up, the termination date will be the date of last contact with the subject.

The end of study evaluations will include the following:

- Collect oral azacitidine study medication (if last dose of study drug was oral azacitidine);
- Diary card review (if last dose of study drug was oral azacitidine);
- Document concomitant medications and transfusions;
- AE assessment (for AEs that occurred within 28 days following the last dose of study drug);
- Physical examination;
- Vital signs, including systolic/diastolic blood pressure, pulse rate, respiratory rate, weight, and oral temperature;
- Hematology and serum chemistry as defined in Section 6.2.3.2;
- Coagulation evaluations, ie, International Normalized Ratio (INR), prothrombin time (PT), and partial thromboplastin time (PTT);
- Serum pregnancy test – if applicable for females of childbearing potential;
- 12-lead ECG;
- ECOG Performance Score;
- Revised IWG hematologic response and/or improvement assessment (see Appendix D, Section **Error! Reference source not found.**; Appendix I, Section **Error! Reference source not found.**; and Appendix J, Section **Error! Reference source not found.**).
- Bone marrow aspirate (if early withdrawal and not collected within the previous 7 days), CBC and PD blood sampling; and
- Cytogenetic testing on bone marrow aspirate if collected.

Subjects discontinuing from the OEP will have an OEP discontinuation visit 28 days after the last dose of study drug or at study withdrawal, even if the date does not correspond to a protocol-specified visit.

Refer to Appendix M, Section **Error! Reference source not found.** for End of Study/Discontinuation Procedures in the OEP.

5.4. Additional Follow-Up

Follow-up evaluations will be performed for clinically significant abnormal physical examinations or abnormal laboratory findings, 12-lead ECGs, or vital signs, as deemed necessary by the Investigator. Pharmacodynamic blood and bone marrow sampling should accompany clinically indicated/unscheduled procedures. All subjects who have adverse events, whether considered associated with the use of the investigational product or not, must be monitored to determine the outcome. The clinical course of the adverse event will be followed according to accepted standards of medical practice, even after the end of the period of observation, until a satisfactory explanation is found or the Investigator deems it medically justifiable to terminate follow-up. Should the adverse event result in death, a full pathologist's report should be supplied.

5.5. Optional Extension Phase

Upon a site's IRB approval of protocol Amendment 5 which allows for an OEP, any subject enrolled in Part 1 or Part 2 continuing to receive oral azacitidine who has stable disease or is demonstrating clinical benefit as assessed by the Investigator, and has consented to participate in Amendment 5, may enter the OEP of this study (at their current dose) at the start of their next cycle.

Subjects may continue to receive oral azacitidine in the OEP until they meet the criteria for study discontinuation (Section **Error! Reference source not found.**) or until the availability of a rollover protocol exists, into which any subjects who remain on study may be consented and continue to receive treatment with oral azacitidine. It must be the opinion of the investigator that the remaining subject(s) continue to receive benefit from treatment with oral azacitidine. Details for the OEP are provided in Appendix M, Section **Error! Reference source not found.**.

6. DATA MEASUREMENTS AND METHODS OF COLLECTION

6.1. Data Measurements

6.1.1. Safety Data

Safety variables for this study include the following:

- DLTs (during the first cycle of oral azacitidine treatment only);
- Physical examination;
- Vital signs;
- 12-lead ECG;
- Clinical laboratory measurements, including hematology, serum chemistry, and coagulation tests; and pregnancy tests
- Adverse events.

6.1.2. Pharmacokinetic Data

Multiple PK parameters will be estimated using actual elapsed time from dosing. Table 1 and Table 2 show the plasma PK parameters to be determined after single and multiple oral dose(s), respectively. Table 3 lists the urine PK parameters of interest.

Table 1: Pharmacokinetic Parameters Following Single Oral Administration

C_{max} (ng/mL)	Observed maximum plasma concentration
t_{max} (h)	Observed time of maximum plasma concentration
λz (h^{-1})	Elimination rate constant, determined by linear regression of the terminal points of the log-linear plasma concentration-time curve. Visual assessment will be used to identify the terminal linear phase of the concentration-time profile. A minimum of 3 data points will be used for determination (C_{max} excluded)
$t_{½}$ (h)	Terminal elimination half-life, determined from the quotient 0.693 / □λz
$AUC_{(0-t)}$ (ng*h/mL)	Area under the plasma concentration-time curve from time zero to time t, calculated by log-linear trapezoidal summation
$AUC_{(0-\infty)}$ (ng*h/mL)	Area under the plasma concentration-time curve from time zero to infinity, calculated by log-linear trapezoidal summation and extrapolated to infinity by addition of the last observed quantifiable plasma concentration divided by the elimination rate constant □z (if % AUC_{ext} > 20%, $AUC_{(0-\infty)}$ will not be reported)
$AUC_{ext\%}$	The percent of the area under the concentration-time curve which was extrapolated to infinity (%), calculated as ($C_{last}/\lambda z$) / $AUC_{(0-\infty)}$ *100.
CL_{oral} (L/h)	Apparent clearance after oral administration, calculated from the quotient dose / $AUC_{(0-\infty)}$

Table 2: Pharmacokinetic Parameters Following Multiple Oral Administration

Cmax,ss (ng/mL)	Observed maximum plasma concentration at steady state
tmax,ss (h)	Observed time of maximum plasma concentration at steady state
λz (h-1)	Elimination rate constant, as defined above
t½ (h)	Terminal elimination half-life, as defined above
AUC(0-t),ss (ng*h/mL)	Area under the plasma concentration-time curve from time zero to time t at steady state, as defined above
Cmax AR	Cmax accumulation ratio, calculated as Cmax,ss / Cmax
AUC AR	AUC accumulation ratio, calculated as AUC(0-t),ss / AUC(0-t)

The following azacitidine urine PK parameters will be estimated:

Table 3: Urine Pharmacokinetic Parameters

$Ae_{(0\text{-}Tlast)}$	Cumulative amount excreted from time zero to time T_{last} (ng), calculated as the summation of the amounts (Ae_t, product of urine volume and urine concentration) excreted in subsequent collection intervals.
$fe_{(0\text{-}Tlast)}$	Cumulative fraction of dose excreted in the urine from time zero to time T_{last} (%), calculated as $Ae_{(0\text{-}Tlast)}$ divided by dose.
CLr	Renal clearance (mL/min), calculated as $Ae_{(0\text{-}Tlast)}$ divided by $AUC_{(0\text{-}Tlast)}$.

6.1.3. Correlative Studies Data (Pharmacodynamic)

Possible relationships between PD variables, PK parameters, and clinical outcome may be explored, if appropriate.

Pharmacodynamic variables of interest include, but are not limited to changes in:

- Global and gene-specific DNA methylation, gene expression, and levels of fetal hemoglobin and soluble (biomarker) factors after azacitidine administration; and
- Potential stratification or predictive markers of interest include: occurrence of SNPs in genes relevant to azacitidine metabolism and soluble factors in plasma (biomarkers).

6.1.4. Efficacy Data

The efficacy endpoint will be hematologic response, defined as CR, PR, SD, or hematologic improvement for subjects with MDS or CMML and CR or PR for subjects with AML based on IWG criteria (see Appendix D, Section **Error! Reference source not found.**; Appendix I, Section **Error! Reference source not found.**; and Appendix J, Section **Error! Reference source not found.**). Subjects with CMML will be assessed for response according to the IWG criteria for MDS. The efficacy variables, which will be used to assess response, include hemoglobin, ANC, platelets, bone marrow aspirate/biopsy, and transfusions.

6.2. Methods of Collection

6.2.1. Pharmacokinetic Sample Collection

Note: All PK sampling for Part 2 subjects will be discontinued with implementation of Amendment #4. All Part 1 subjects have completed PK sampling.

6.2.1.1. Blood Samples

Blood samples (3 mL) will be collected from each subject for the determination of plasma azacitidine concentrations by in-dwelling catheter or by venipuncture into sample collection tubes as described in the Laboratory Manual (to be supplied). If the SC injection is given in an arm, collect pharmacokinetic blood samples from the opposite arm.

6.2.1.1.1. Part 1

Days and timing of pharmacokinetic blood sampling will be as follows:

- Cycle 1: on Days 1 and 7 prior to dosing (within 1 hour prior to dosing) and at the following post-dose times: 0.25, 0.5, 1, 1.5, 2, 2.5, 3, 3.5, 4, 6, and 8 hours;
- Cycle 2: on Days 1 and 7 prior to dosing (within 1 hour prior to dosing) and at the following post-dose times: 0.25, 0.5, 1, 1.5, 2, 2.5, 3, 3.5, 4, 6, and 8 hours;
- Cycle 7: on Day 7 prior to dosing (within 1 hour prior to dosing) and at the following post-dose times: 0.25, 0.5, 1, 1.5, 2, 2.5, 3, 3.5, 4, 6, and 8 hours.

6.2.1.1.2. Part 2

Days and timing of pharmacokinetic blood sampling will be as follows:

- Cycle 1: on Day 1 (all subjects) and either Day 14 or Day 21, depending on the subject's treatment schedule, prior to dosing (within 1 hour prior to dosing) and at the following post-dose times: 0.25, 0.5, 1, 1.5, 2, 2.5, 3, 3.5, 4, 6, and 8 hours;
- Cycle 6: on Day 14 or Day 21, depending on the subject's treatment schedule, prior to dosing (within 1 hour prior to dosing) and at the following post-dose times: 0.25, 0.5, 1, 1.5, 2, 2.5, 3, 3.5, 4, 6, and 8 hours.

Subjects on a BID treatment schedule will have PK samples drawn before and following the first dose taken on Day 1 and before and following the first dose taken on either Day 14 or Day 21, depending on the subject's treatment schedule.

The label on each sample tube will state the Sponsor name, study number, site number, subject number, visit name, sample type, date, and time. The exact date and time of each sample collection will also be recorded in the CRF.

Azacitidine is unstable in blood and plasma; all blood samples will be processed and plasma harvested immediately per the instructions in the Laboratory Manual. In general, these procedures will involve collection of blood using pre-chilled vacutainers; gently mixing blood with a preservative and anticoagulant then immediately placing the vacutainer of freshly collected blood into an ice bath. The blood will then be centrifuged using a refrigerated (4ºC) centrifuge for 5 to 10 minutes at approximately 2,000 x g, followed by immediate harvest of the plasma and storage in an ultra-low temperature freezer at -70ºC or colder.

All plasma samples will be kept frozen at -70ºC or colder and shipped in dry ice according to instructions provided in the Laboratory Manual. Analysis of plasma samples for azacitidine will be performed using a high performance liquid chromatography/tandem mass spectrometry (LC-MS/MS) method specifically validated for the determination of azacitidine in plasma.

A maximum total of 60 samples (approximately 180 mL of blood) over the course of 7 cycles will be collected from each subject in Part 1 for pharmacokinetic analysis. A maximum total of 36 samples (approximately 108 mL of blood) over the course of 6 cycles will be collected from each subject in Part 2 for pharmacokinetic analysis.

6.2.1.2. Urine Samples

In Part 1, pharmacokinetic urine samples will be collected during Cycles 1 and 2 on Days 1 and 7. All voided urine will be collected separately beginning with a predose sample (within 1 hour prior to dosing), and thereafter up to 8 hours following azacitidine administration. The date, time, and volume of each void will be recorded in the CRF. Two aliquots will be frozen (see Laboratory Manual) and the remainder discarded.

The label on each sample aliquot will state the Sponsor name, study number, site number, subject number, visit name, sample type, and exact date and time. The exact date and time of each sample collection will be recorded in the CRF, as well as the exact total void volume.

All urine samples will be kept frozen at -70ºC or colder and shipped in dry ice according to instructions provided in the Laboratory Manual. Analysis of urine samples for azacitidine will

be performed using a LC-MS/MS method specifically validated for the determination for azacitidine in urine.

6.2.2. Sample Collection for Correlative Studies (Pharmacodynamics)

6.2.2.1. Blood Samples

6.2.2.1.1. Part 1

In order to evaluate potential PD effects of SC and/or oral azacitidine, samples of whole blood will be obtained using standard phlebotomy procedures at the following time points:

- Screening, same day as the bone marrow procedure, no more than 7 days prior to Cycle 1, Day 1;
- Cycle 1, Day 3 (pre-dose);
- Cycle 1, Day 8 (± 3 days) with the bone marrow procedure;
- Cycle 1, Day 15;
- Cycle 1, Day 22;
- Cycle 1, Day 28 or within 7 days of Cycle 2, Day 1 with the bone marrow procedure;
- Cycle 2, Day 3 (pre-dose);
- Cycle 2, Day 8 (± 3 days);
- Cycle 2, Day 15;
- Cycle 2, Day 22;
- Cycles 3, 4, 5 and 6, Day 1 (pre-dose);
- Cycles 3, 4, 5 and 6, Day 15;
- Cycle 7, Day 1 (pre-dose);
- Cycle 7, Day 7 (pre-dose);
- Cycle 7, Day 28 or within 7 days of Cycle 8, Day 1 with the bone marrow procedure;
- Cycles 8 and Beyond: Oral Azacitidine, Day 1 (pre-dose);
- Cycles 8 and Beyond: SC Azacitidine, Day 1 (pre-dose);
- Any time a bone marrow procedure is conducted; and
- At the end of the study with the bone marrow procedure.

No more than 25 mL will be collected at each sampling. Whole and fractionated blood samples (plasma and white blood cells) will be stored frozen (-70°C or colder) until shipped on dry ice to the Sponsor or a Sponsor-designated laboratory. No more than 525 mL of whole blood will be collected for PD assessment from each subject in Part 1 over the course of 7 cycles.

6.2.2.1.2. Part 2

In order to evaluate potential PD effects of oral azacitidine, samples of whole blood will be obtained using standard phlebotomy procedures at the following time points:

- Screening, same day as the bone marrow procedure, no more than 21 days prior to Cycle 1, Day 1;
- Cycle 1, Day 1 (pre-dose);
- Cycle 1, Day 8 (± 1 day);
- Cycle 1, Day 14 (± 1 day);
- Cycle 1, Day 21 (± 2 days) with the bone marrow procedure;
- Cycle 2, Day 1 (pre-dose);
- Cycle 2, Day 8 (± 1 day);
- Cycle 2, Day 14 (± 1 day);
- Cycle 2, Day 21 (± 2 days) with the bone marrow procedure;
- Cycle 3, Day 1 (pre-dose);
- Cycle 4, Day 1 (pre-dose);
- Cycle 5, Day 1 (pre-dose) with the Cycle 4, Day 28 bone marrow procedure;
- Cycle 6, Day 1 (pre-dose);
- Cycles 7 and Beyond: Oral Azacitidine, Day 1 (pre-dose) (Cycle 7, Day 1 with the Cycle 6, Day 28 bone marrow procedure);
- Cycles 7 and Beyond: SC Azacitidine, Day 1 (pre-dose) (Cycle 7, Day 1 with the Cycle 6, Day 28 bone marrow procedure);
- Any time a bone marrow procedure is conducted; and
- At the end of the study with the bone marrow procedure.

No more than 25 mL will be collected at each sampling. Whole and fractionated blood samples (plasma and white blood cells) will be stored frozen (-70°C or colder) until shipped on dry ice to the Sponsor or a Sponsor-designated laboratory. No more than 325 mL of whole blood will be collected for PD assessment from each subject in Part 2 over the course of 6 cycles.

6.2.2.2. Bone Marrow Aspirate Samples

In the event that an aspirate cannot be obtained, a bone marrow biopsy should be performed. Biopsy material not used for response and/or other clinical assessments should be made available for PD assessment. All sample handling and shipping information is described in detail in a separate Laboratory Manual (to be supplied).

6.2.2.2.1. Part 1

Bone marrow will be collected using standard institutional procedures at screening (baseline sample will be obtained no more than 7 days prior to Cycle 1, Day 1), and on Cycle 1, Day 8 (± 3 days) following administration of SC azacitidine. To compare the effects of SC and oral azacitidine within each subject, bone marrow aspirates will also be collected before and after administration of oral azacitidine (no more than 7 days prior to Cycle 2, Day 1; and Cycle 2, Day 8 ± 3 days, respectively. Additional aspirates should be obtained during Cycle 7 on Day 28 (with a ± 7 day window) and at the end of study to evaluate the duration of changes, if any, in the bone marrow. A portion of each aspirate (approximately 6 to 8 mL) should be prepared and stored as described in the Laboratory Manual (to be supplied) for shipment to the Sponsor or a Sponsor-designated laboratory for PD analysis. Cytogenetics will be determined on aspirates collected at screening, during Cycle 7, at end of study, and whenever clinically indicated.

6.2.2.2.2. Part 2

Bone marrow will be collected using standard institutional procedures at screening (baseline sample will be obtained no more than 21 days prior to Cycle 1 Day 1), and on Cycle 1, Day 21 (± 2 days) following administration of oral azacitidine. Additional aspirates should be obtained on Cycle 2, Day 21 (± 2 days), Cycle 4, Day 28 (± 3 days), Cycle 6, Day 28 (± 3 days), and at the end of study to evaluate the duration of changes, if any, in the bone marrow. A portion of each aspirate (approximately 6 to 8 mL) should be prepared and stored as described in the Laboratory Manual (to be supplied) for shipment to the Sponsor or a Sponsor-designated laboratory for PD analysis. Cytogenetics will be determined on aspirates collected at screening, at the end of Cycle 6, at end of study, and whenever clinically indicated.

6.2.3. Safety Measurements

Safety measurements for this study include AEs, clinical laboratory measurements (hematology and serum chemistry), physical examination findings, vital signs, and 12-lead ECGs.

6.2.3.1. Adverse Events

Adverse events that occur prior to dosing will be collected on Day 1 by questioning the subject on the occurrence of events since the screening visit. Adverse events will be collected at each clinic visit based on observation and spontaneous reporting.

6.2.3.2. Clinical Laboratory Measurements

All clinical laboratory assessments for Part 1, Part 2 and the OEP of the study are detailed in **Error! Reference source not found.**, **Error! Reference source not found.**, **Error! Reference source not found.**, **Error! Reference source not found.**, **Error! Reference source not found.** and Section 5.

For subjects in Part 1, a hematology assessment will be performed at screening, Cycles 1 and 2, Days 1, 8, 15, 22 and 28, Cycles 3 and beyond, Days 1, 15, and 28, and end of study. For subjects in Part 2, a hematology assessment will be performed at screening, Cycles 1 and 2, Days 1, 8, 14, 21 and 28, Cycles 3 and beyond, Days 1, 14 and 28, and end of study. For subjects in the OEP, a hematology assessment will be performed at Day 1 of every cycle. The hematology assessments are to include:

- Complete blood count including manual WBC absolute differential and platelet count; and
- Red blood cell indices (MCV, MCH, and MCHC).

For subjects in Part 1, a serum chemistry assessment will be performed at screening, Cycles 1 and 2, Days 1 and 28, Cycles 3 and beyond, Day 28, and end of study. For subjects in Part 2, a serum chemistry assessment will be performed at screening, Cycles 1 and 2, Days 1, 14 and 28, Cycles 3 and beyond, Days 14 and 28, and end of study. For subjects in the OEP, a chemistry assessessment will be performed at Day 1 of every cycle. The serum chemistry assessments are to include:

- Electrolytes: (sodium, potassium, calcium, phosphorous, bicarbonate, and chloride)
- Renal function: (BUN, creatinine, and uric acid);
- Liver function: SGOT (also known as AST), SGPT (also known as ALT), total bilirubin, LDH, and alkaline phosphatase;
- Other: (glucose, albumin, and total protein).

Serum vitamin B12 and folate samples are to be collected at screening.

Coagulation testing will be performed at screening and end of study. Coagulation tests are to include INR, PT, and PTT.

Serum pregnancy tests for females of childbearing potential at screening (obtained no more than 7 days prior to dosing on Cycle 1 Day 1) and at end of study and urine or serum pregnancy tests obtained within 7 days prior to dosing on Day 1 of each cycle.

Approximately 152 mL of blood will be collected for safety assessments from each subject in Part 1 over the course of 7 cycles. If the subject remained on oral azacitidine beyond Cycle 7, each additional cycle starting with Cycle 8 would require 4 blood samples (16 mL). In the event the subject crosses over to SC azacitidine, Cycle 8 would require 5 blood samples (20 mL) and each additional cycle starting with Cycle 9 would require 4 blood samples (16 mL). The end of study safety laboratory assessments would require 4 blood samples (16 mL).

Approximately 156 mL of blood will be collected for safety assessments from each subject in Part 2 over the course of 6 cycles. If the subject remained on oral azacitidine beyond Cycle 6, each additional cycle starting with Cycle 7 would require 5 blood samples (20 mL). In the event the subject crosses over to SC azacitidine, Cycle 7 would require 5 blood samples (20 mL) and each additional cycle starting with Cycle 8 would require 4 blood samples (16 mL). The end of study safety laboratory assessments would require 4 blood samples (16 mL).

Abnormal, clinically significant results as assessed by the Investigator should be repeated to rule out laboratory error. Persistent relevant abnormal changes from baseline must be followed up until the cause is determined or until they return to the premedication value. Hematology and serum chemistry will be carried out according to the standard operating procedures by the validated local laboratory.

6.2.3.3. Physical Examination, Vital Signs, and ECG

For Part 1 and Part 2, a physical examination, and 12-lead ECG (rhythm, heart rate, and PR, QRS, and QT intervals) will be conducted at screening, predose on Day 1 of every cycle, and at end of study.

In the OEP, clinical assessment by the Investigator will be performed at clinic visits. This may include physical exam, vital signs, body weight – but data will not be collected in the CRF. However, please record in the source documents. Clinically significant abnormal findings should be recorded as adverse events in the clinical database.

7. DISCONTINUATIONS

7.1. Discontinuation of Subjects

A subject must be discontinued from protocol-prescribed therapy under the following circumstances:

- Consent withdrawal at the subject's own request or at the request of their legally authorized representative;
- Unacceptable toxicity or adverse event as determined by the Investigator and/or subject;
- Significant deviation from inclusion/exclusion criteria, in the opinion of the Investigator;
- If, in the Investigator's opinion, continuation in the study could be detrimental to the subject's well-being;
- If a female subject becomes pregnant; or
- At the specific request of the Sponsor or its authorized representative (for example, if the study is terminated due to failure to demonstrate efficacy or for reasons of subject safety).
- Enrolling in the optional extension phase of this study

Subjects who are entering the OEP should be discontinued from Part 1 or Part 2 protocol prescribed therapy. The reason for discontinuation should be recorded as enrolling in the optional extension phase of the study.

If a female subject or the female partner of a male subject who is required to use defined contraceptive methods becomes pregnant during the study, the female subject or pregnant partner must be followed until the outcome of the pregnancy is known (see Section **Error! Reference source not found.**). Both the detection and the outcome of the pregnancy need to be reported to Pharmacovigilance.

Subjects may also be discontinued from protocol-prescribed therapy per Investigator discretion if the subject fails treatment (defined as relapse after CR, PR, or hematologic improvement, or disease progression per IWG criteria for MDS).

As far as possible, all end of study examinations must be performed on all subjects who receive the study drug but do not complete the study according to protocol. All subjects who discontinue from protocol-prescribed therapy for any of the reasons above will be followed for a period of 28 days following the last day of study drug administration.

7.2. Replacement of Subjects

Subjects in Part 1 who are withdrawn prior to Cycle 2 for any reason will be replaced. For a subject to be considered evaluable in dose-escalation decisions, the subject must have received at least 5 of the 7 scheduled doses and have completed Cycle 2, Day 28 without a DLT, experienced a DLT, or have been withdrawn from the study prior to completing Cycle 2, Day 28

due to a DLT. If a subject withdraws from the study without having met any of these criteria, then the subject will be replaced in that cohort.

For a subject in Part 2 to be considered evaluable in dose-escalation decisions, the subject must have received at least 10 of 14 scheduled doses in the 14-day QD treatment schedule, or at least 20 of 28 scheduled doses in the 14-day BID treatment schedule, or at least 15 of 21 scheduled doses in the 21-day QD treatment schedule, or at least 30 of 42 scheduled doses in the 21-day BID treatment schedule, and have completed Cycle 1, Day 28 without a DLT, or have experienced a DLT. If a subject withdraws from the study without having met any of these criteria, then the subject will be replaced in that cohort.

7.3. Study Stopping Rules

To reduce the risk of exposing subjects to an excessively toxic dose level, a stopping rule will be implemented if more than 33% of subjects in a given oral azacitidine MTD or treatment schedule expansion arm experience a DLT. For example, if after 12 subjects are enrolled and treated, 5 are found to have experienced a DLT, then enrollment into that MTD or treatment schedule expansion arm will stop. At least 12 subjects should be enrolled into an MTD or treatment schedule expansion arm before this rule will take effect.

Should an MTD or treatment schedule expansion arm be terminated prematurely due to safety concerns, a lower dose level may be considered for further evaluation.

To ensure subject safety in the dose-escalation portion of the study, an early stopping rule will be implemented in the event that a DLT rate of greater than 33% is observed at the lowest dose level to be evaluated in a treatment schedule. In the event a DLT rate of greater than 33% is observed at the starting dose in a treatment schedule, then the dose will be reduced and additional subjects evaluated.

8. STATISTICAL METHODS

The sections below provide an overview of the proposed statistical considerations and analyses. The final statistical analysis methods will be documented in detail in the Statistical Analysis Plan.

8.1. Determination of Sample Size

No formal sample size calculations were performed. It is estimated that up to 150 subjects will be enrolled in this study. Precise sample size cannot be defined, as it is dependent on the observed toxicity rate. A description of the statistical characteristics (probabilities of halting or continuing dose escalation) of the "3 + 3" design in Part 1 is provided in Appendix K, Section **Error! Reference source not found.**. A description of the statistical characteristics for enrollment of 6 subjects per cohort in Part 2 of the study is provided in Appendix L, Section **Error! Reference source not found.**. Cohorts of 3 to 6 subjects will be treated at each oral azacitidine dose level in a treatment schedule until the MTD for that schedule is reached. In Part 1, sequential cohorts of subjects will be enrolled until the MTD is reached. In Part 2, cohorts of subjects will be enrolled sequentially or in parallel in the different treatment schedules until the MTD in each schedule is reached. It is anticipated that up to 20 subjects in total may be evaluated at the MTD of a given treatment schedule. The exposure of additional subjects at the MTD in a treatment schedule will provide a better estimate of the toxicity rate. In the event that MTD is not reached on one or more treatment schedules and/or the Investigators and Sponsor's Medical Monitor agree that further dose escalation on one or more treatment schedules is not warranted, approximately 20 low and/or Int-1 risk MDS subjects, not inclusive of those treated prior to implementation of Amendment #4, may be enrolled and treated at a previously evaluated dose on a given treatment schedule and/or at a lower dose than previously evaluated on that schedule. Expanded treatment schedules may be evaluated concurrently or sequentially as agreed upon between the Investigators and Sponsor's Medical Monitor.

8.2. Analysis Populations

8.2.1. Safety Population

The safety population will consist of all subjects who received at least 1 dose of study drug and had at least 1 post-dose safety assessment. Safety summaries will include all subjects in the safety population.

8.2.2. Dose-Limiting Toxicity Evaluable Population

A subject in Part 1 is evaluable in dose-escalation decisions provided the subject has received at least 5 of the 7 scheduled doses and has completed Cycle 2, Day 28 without a DLT, has experienced a DLT, or has been withdrawn from the study prior to completing Cycle 2, Day 28 due to a DLT. If a subject withdraws from the study without meeting these criteria, the subject will be replaced in that cohort. Tabulations of DLTs will only include the DLT-evaluable population. Subjects in Part 1 withdrawn during Cycle 1 will not be eligible for dose escalation decisions.

A subject in Part 2 is evaluable for dose-escalation decisions provided the subject has received at least 10 of 14 scheduled doses in the 14-day QD treatment schedule, or at least 20 of 28

scheduled doses in the 14-day BID treatment schedule, or at least 15 of 21 scheduled doses in the 21-day QD treatment schedule, or at least 30 of 42 scheduled doses in the 21-day BID treatment schedule, and has completed Cycle 1, Day 28 without a DLT, or has experienced a DLT. If a subject withdraws from the study without meeting these criteria, the subject will be replaced in that cohort. Tabulations of DLTs will only include the DLT-evaluable population.

8.2.3. Pharmacokinetic Population

The pharmacokinetic population will consist of all subjects who had sufficient concentration-time data to enable the calculation of pharmacokinetic parameters for azacitidine for at least 1 cycle. For subjects that were determined to be noncompliant with respect to administration of azacitidine, or for subjects with incomplete data, a decision as to their inclusion in the analysis will be made on a case-by-case basis.

8.2.4. Pharmacodynamic Population

The pharmacodynamic population will consist of all subjects with any evaluable PD results.

8.2.5. Efficacy Evaluable Population

The efficacy population will include all subjects who receive at least 1 dose of study drug and had at least 1 postdose efficacy assessment.

8.3. Analysis Methods

8.3.1. Safety

Safety evaluation will include monitoring for adverse events, scheduled laboratory assessments, vital sign measurements, ECGs, and physical examinations. The intensity of adverse changes in physical signs or symptoms will be graded according to the CTCAE version 3.0 (see Study Reference Manual). For all other adverse events not listed in the CTCAE, the intensity of these events will be assessed by the Investigator using a 5-point scale as described in Section **Error! Reference source not found.**.

Adverse events will be coded in the Medical Dictionary for Regulatory Activities (MedDRA) and listings will be prepared that include the verbatim term, preferred term, and system organ class (SOC). The number of adverse events and the incidence of adverse events by SOC and preferred term will be summarized. Adverse events will be summarized by maximum intensity (as described in Section **Error! Reference source not found.**) and relationship to study drug for each treatment group. Separate summaries will be provided for all adverse events, serious adverse events, treatment-related adverse events, and other significant adverse events (eg, adverse events leading to study discontinuation).

Clinical laboratory results will be listed by subject or, as appropriate, summarized descriptively by treatment group, which will include a display of change from baseline. Laboratory values outside of the normal ranges will be identified. Clinically significant hematologic laboratory abnormalities (ie, meet Grade 3, 4, or 5 criteria according to CTCAE) will be listed and summarized.

Physical examination, vital sign, and ECG data will be listed for each subject at each visit. If appropriate, descriptive statistics for vital signs, both observed values and changes from baseline, will be summarized by treatment group.

8.3.2. Pharmacokinetics

The pharmacokinetic analysis will be done using noncompartmental methods which will be documented in detail in the Statistical Analysis Plan.

By-subject listing of pharmacokinetic blood sample collection times as well as derived sampling time deviations will be provided. Azacitidine plasma concentrations will be summarized using descriptive statistics (N, arithmetic mean, standard deviation, minimum, median, maximum, percent coefficient of variation, and geometric mean) for each treatment. Concentrations that are below the limit of quantitation (BLQ) will be treated as zero for the computation of descriptive statistics and listed with the lower limit of quantitation (LLQ) indicated. Missing concentrations will be omitted from the calculation of descriptive statistics.

Figures of mean azacitidine concentration-time data will also be illustrated for each treatment. Individual azacitidine subject concentration-time data for each treatment will be graphically presented on linear and semi-logarithmic scales.

Following single dose administration, predose samples that are BLQ or missing will be assigned a numerical value of zero for the calculation of AUC. Any anomalous concentration values observed at predose will be identified in the study report and used for the computation of AUC. Pharmacokinetic parameters will be computed if the anomalous value is not greater than 5% of the C_{max}. If the anomalous value is greater than 5% of C_{max}, the computed pharmacokinetic parameters for the given subject will be dropped from the pharmacokinetic analysis.

Any other BLQ concentrations will be assigned a value of zero if they precede quantifiable samples in the initial portion of the profile. A BLQ value that occurs between quantifiable data points, especially prior to C_{max}, will be evaluated to determine if an assigned concentration of zero makes sense, or if reanalysis or exclusion of the data is warranted. Following C_{max}, BLQ values embedded between 2 quantifiable data points will be treated as missing when calculating AUC. BLQ values occurring at the end of the collection interval (after the last quantifiable concentration) will be treated as missing data. If consecutive BLQ concentrations are followed by quantifiable concentrations in the terminal portion of the concentration curve, these quantified values will be excluded from the pharmacokinetic analysis by assigning them a value of missing, unless otherwise warranted by the concentration-time profile. For the purpose of analysis, these trailing BLQ values may be designated as zero in the dataset if the pharmacokinetic program used to do the analysis (such as WinNonlin®) will treat trailing zero values as missing when calculating AUC.

Pharmacokinetic parameters will be derived using noncompartmental methods with WinNonlin® Professional Version 5.1, or higher, (Pharsight Corp., Mountain View, California). All PK computations will be performed using WinNonlin Professional Version 5.1, or higher; Excel 2002, or higher (Microsoft Corp., Seattle, Washington); or SAS® Version 9.1, or higher (SAS Institute, Inc., Cary, North Carolina). Graphics may be prepared with SAS Version 9.1, or higher; or Excel 2002, or higher; or WinNonlin Professional 5.1, or higher.

By-subject listings of PK urine sample collection times will be provided. Urine volumes, azacitidine urine concentrations, and urine PK parameters will be summarized using descriptive statistics (N, arithmetic mean, standard deviation, minimum, median, maximum, percent coefficient of variation, and geometric mean) for each treatment.

8.3.3. Correlative Studies (Pharmacodynamics)

No formal statistical analysis of pharmacodynamic endpoints will be performed. Possible relationships between PK, PD, safety and efficacy variables will be explored, as appropriate.

8.3.4. Efficacy

All efficacy endpoints will be summarized descriptively using frequency distributions. In addition, 95% confidence intervals will be provided for the proportion of each response/improvement.

8.4. Analysis from OEP

Please refer to Section **Error! Reference source not found.** for analysis to be performed for the OEP.

9. REFERENCES

Celgene Corporation. Azacitidine Investigator's Brochure, Edition 7. Summit, NJ; Jun 17 2010.
