# Supplementary material for: Pharmacokinetics and Pharmacodynamics with Extended Dosing of CC-486 in Patients with Hematologic Malignancies
Source: PLoS One. 2015 Aug 21;10(8):e0135520. doi: 10.1371/journal.pone.0135520 (PMC4546409; doi:10.1371/journal.pone.0135520)
Supplement: S1 Table — (DOCX) [file pone.0135520.s005.docx]

1. List of IEC’s or IRB’s and Representative Written Information for Patient and Sample Consent Forms

**List of IECs/IRBs for AZA PH US 2007 CL 005**

| Site No. | Investigator Name | Name/Address of IEC/IRB | Name of Chairperson(s) | **IRB approval Initial** |
| --- | --- | --- | --- | --- |
| 01 | Christopher Cogle, MD | Western Institutional Review Board (WIRB)  3535 Seventh Avenue SW  Olympia, WA 98508-5010 | R. Bert Wilkins | 5/29/08 |
| 02 | Guillermo Garcia-Manero, M.D. | The University of Texas M.D. Anderson Cancer Center  Surveillance Committee - FWA 363  1515 Holcombe Boulevard - Box 038  Houston, TX 77030-4009 | FWA00000363 | 8/1/07 |
| 03 | Amy DeZern  Steven D. Gore, MD (former PI) | Johns Hopkins Medicine Institutional Review Boards  Reed Hall B-130  1620 McElderry Street  Baltimore, MD 21205-1922 | Kenneth Cohen | 10/27/09 |
| 04 | Suman Kambhampati, M.D.  Barry S. Skikne, MD (former PI) | Human Subjects Committee  The University of Kansas Medical Center  3901 Rainbow Blvd.  Kansas City, KS 66160 | FWA00003411 | 7/30/07 |
| 06 | Olatoyosi Odenike, MBBS | The University of Chicago  Institutional Review Board  McGiffert Hall, 2nd Floor  5751 S. Woodlawn Ave.  Chicago, IL 60637 | FWA00005565 | 10/11/11 |
| 08 | Roger M. Lyons, MD | Western Institutional Review Board (WIRB)  3535 Seventh Avenue SW  Olympia, WA 98508 | R. Bert Wilkins | 3/18/11 |
| 09 | Bart L. Scott, M.D. | Karen Hansen, Administrator  Institutional Review Board  Fred Hutchinson Cancer Research Center  1100 Fairview Avenue North  P.O. Box 19024  Seattle, WA 98109-1024 | FWA00000021 | 2/9/11 |
| 10 | Suman Kambhampati, M.D. | Kansas City Veterans Affairs Medical Center IRB  4801 Linwood Blvd.  Kansas City, MO 64128 | FWA00001481 | 3/3/11 |
| 11 | Thomas E. Boyd, MD | US Oncology Inc.  Institutional Review Board  10101 Woodloch Forest Dr.  The Woodlands, TX 77380 | IORG0000759 | 1/6/11 |
| 13 | Paul R. Conkling, MD | US Oncology Inc.  Institutional Review Board  10101 Woodloch Forest Dr.  The Woodlands, TX 77380 | IORG0000759 | 1/6/11 |
| 14 | Lawrence E. Garbo, MD | US Oncology Inc.  Institutional Review Board  10101 Woodloch Forest Dr.  The Woodlands, TX 77380 | IORG0000759 | 1/6/11 |
| 16 | William J. Edenfield, MD | US Oncology Inc.  Institutional Review Board  10101 Woodloch Forest Dr.  The Woodlands, TX 77380 | IORG0000759 | 1/6/11 |
| 17 | Edwin C. Kingsley, MD | US Oncology Inc.  Institutional Review Board  10101 Woodloch Forest Dr.  The Woodlands, TX 77380 | IORG0000759 | 1/6/11 |
| 21 | Ayalew Tefferi, M.D. | Mayo Clinic Institutional Review Board  200 First Street, Southwest  201 Building, Room 4-60  Rochester, MN 55905 | FWA00005001 | 8/24/11 |
| 23 | Michael R. Savona, MD | Integ Review Ethical Review Board  3001 S. Lamar Blvd., Ste 210  Austin, TX 78704 | IORG0000689 | 8/30/11 |
