# Supplementary material for: Pharmacokinetics and Pharmacodynamics with Extended Dosing of CC-486 in Patients with Hematologic Malignancies
Source: PLoS One. 2015 Aug 21;10(8):e0135520. doi: 10.1371/journal.pone.0135520 (PMC4546409; doi:10.1371/journal.pone.0135520)
Supplement: S3 Table — Kinetics of mean methylation changes in the top 5 hypomethylated loci are shown in S3 Fig. (DOC) [file pone.0135520.s007.doc]

**Supplementary Table 2. Hypomethylated loci on day 22 for the CC-486 300 mg once-daily 21-day dosing schedule.** *Kinetics of mean methylation changes in the top 5 demethylated loci are shown in *Supplementary Figure 4*.
